# A Gnawing Question: How Do Caribou and Other Arctic Mammals Exploit Shared Bone Resources?

**DOI:** 10.1002/ece3.72444

**Published:** 2026-02-24

**Authors:** Madison Gaetano, Eric Wald, Patrick Druckenmiller, Joshua H. Miller

**Affiliations:** ^1^ Department of Geosciences University of Cincinnati Cincinnati Ohio USA; ^2^ National Park Service Fairbanks Alaska USA; ^3^ University of Alaska Museum Fairbanks Alaska USA

**Keywords:** caribou ( *Rangifer tarandus* ), female antlers, migratory fidelity, osteophagy, resource partitioning

## Abstract

Bones of dead animals are consumed by many species, yet the partitioning of this resource, and the associated ecological and evolutionary implications, remains poorly understood. Using bone modification features found on shed female caribou ( *Rangifer tarandus* ) antlers and skeletal bones lying on caribou calving grounds of the Arctic National Wildlife Refuge (Alaska), we evaluated resource partitioning by co‐occurring ungulates, carnivorans, and rodents. We found that 86.4% of shed antlers were modified by animals and that caribou were the dominant modifiers (99%); rodent (3.5%) and carnivoran (0%) modifications were rarely observed. Conversely, 44.2% of skeletal bones showed modifications, most of which were attributable to carnivorans (91.9%), and only rarely to caribou (12.1%) and rodents (1%). Carnivoran preferences for skeletal bones over shed antlers are consistent with their proclivity for the bones of recently dead animals, which are rich in fats and associated soft tissues. Ubiquitous ingestion by caribou of their population's shed antlers indicates the importance of a rarely recognized nutrient resource during the calving and post‐calving intervals and offers new insights into the biological benefits of female caribou antlers. Caribou are the only cervid for which females grow antlers. In migratory populations, females shed their antlers after reaching their calving grounds and within only days of birthing their young. Pervasive antler consumption by caribou suggests that synchroneity between birthing and antler shedding evinces the importance of nutrient (Ca, P) transport for supporting calf survival and that osteophagy may have contributed to the evolution and maintenance of antlers in female caribou, along with their peculiar shedding schedules. Antler accumulations may also contribute to calving ground fidelity by establishing a predictable mineral reservoir in nutrient‐poor settings.

## Graphical abstract

Bones of dead animals are consumed by many species, yet the partitioning of this resource, and the associated ecological and evolutionary implications, remains poorly understood. Using bone modification features found on shed female caribou ( *Rangifer tarandus* ) antlers and skeletal bones lying on caribou calving grounds of the Arctic National Wildlife Refuge (Alaska), we evaluated resource partitioning by co‐occurring ungulates, carnivorans, and rodents. Pervasive antler consumption by caribou suggests that synchroneity between birthing and antler shedding evinces the importance of nutrient (Ca, P) transport for supporting calf survival, and that osteophagy may be an important evolutionary driver of female caribou antlers.

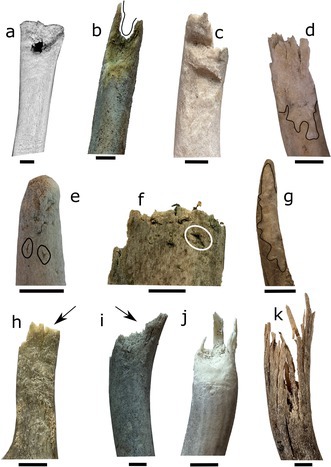

## 1 Introduction

Bones of dead animals lying on landscapes are used by many mammals as an important, though often overlooked, nutritional supplement; particularly when taxa experience dietary deficiencies in calcium, phosphorus and other minerals (Sutcliffe 1973; Brothwell 1976; Pobiner 2008; Hutson et al. 2013). While bone modification is often considered a hallmark of carnivorans and can be highly destructive, such marks frequently occur as collateral damage during the ingestion of soft tissues (Haynes 1983; Blumenschine 1988; Faith and Behrensmeyer 2006; Pobiner 2008). Ungulates, on the other hand, directly target and consume somewhat weathered and dried bones (osteophagy) for their mineral resources (e.g., calcium, sodium, and phosphorus; Wika 1982; Bowyer 1983; Barrette 1985; Van Valkenburgh 1996; Cáceres et al. 2011; Gambín et al. 2017; Rassadnikov 2021). This targeting is similar to rodents, for which bone gnawing has been attributed to both nutritional and biomechanical benefits, including maintaining appropriate lengths of their ever‐growing incisors (Carlson 1940; Olsen 1989; Rinaldi and Cole 2004; Klippel and Synstelien 2007). While bones have broad ecological and nutritional value across mammals, many past studies have focused on the behaviors and bone modifications of single species or discriminating marks made by species within a taxonomic group (e.g., carnivorans, rodents, ungulates; Sutcliffe 1973; Klippel and Synstelien 2007; Pobiner et al. 2007; Fosse et al. 2012; Arilla et al. 2019; Rosell et al. 2019). As such, there are limited data on how mammals partition this resource at community scales (but see discussion of partitioning by carnivorans in Haynes 1982, 1983). Additionally, the capacity of osteophagy to drive aspects of mammal ecology and evolution has been poorly explored (but see Wika 1982; Gambín et al. 2017; Mori et al. 2018).

Multiple lines of evidence indicate that ungulates have the potential to be the dominant modifiers and consumers of bones, relative to other large‐bodied mammals. First, the abundances of ungulates can be orders of magnitude larger than co‐occurring carnivorans (Sinclair et al. 2003; Gervasi et al. 2012). Second, while bone modifications made by mammalian predators and scavengers generally occur within a restricted period following death (Haynes 1983), ungulates will gnaw on bones well after those bones first appeared on a landscape and have weathered (Cáceres et al. 2011; Hutson et al. 2013). However, while ungulates may play important roles in bone modification and recycling, they are rarely the focus of taphonomic studies (but see Sutcliffe 1973; Wika 1982; Cáceres et al. 2011; Hutson et al. 2013; Gambín et al. 2017; Rassadnikov 2021). Due to this investigative gap, the overall impacts of ungulates on regional bone accumulations are largely unknown, as is the importance of bone as a consumable nutrient resource for ungulates.

**FIGURE 1:**
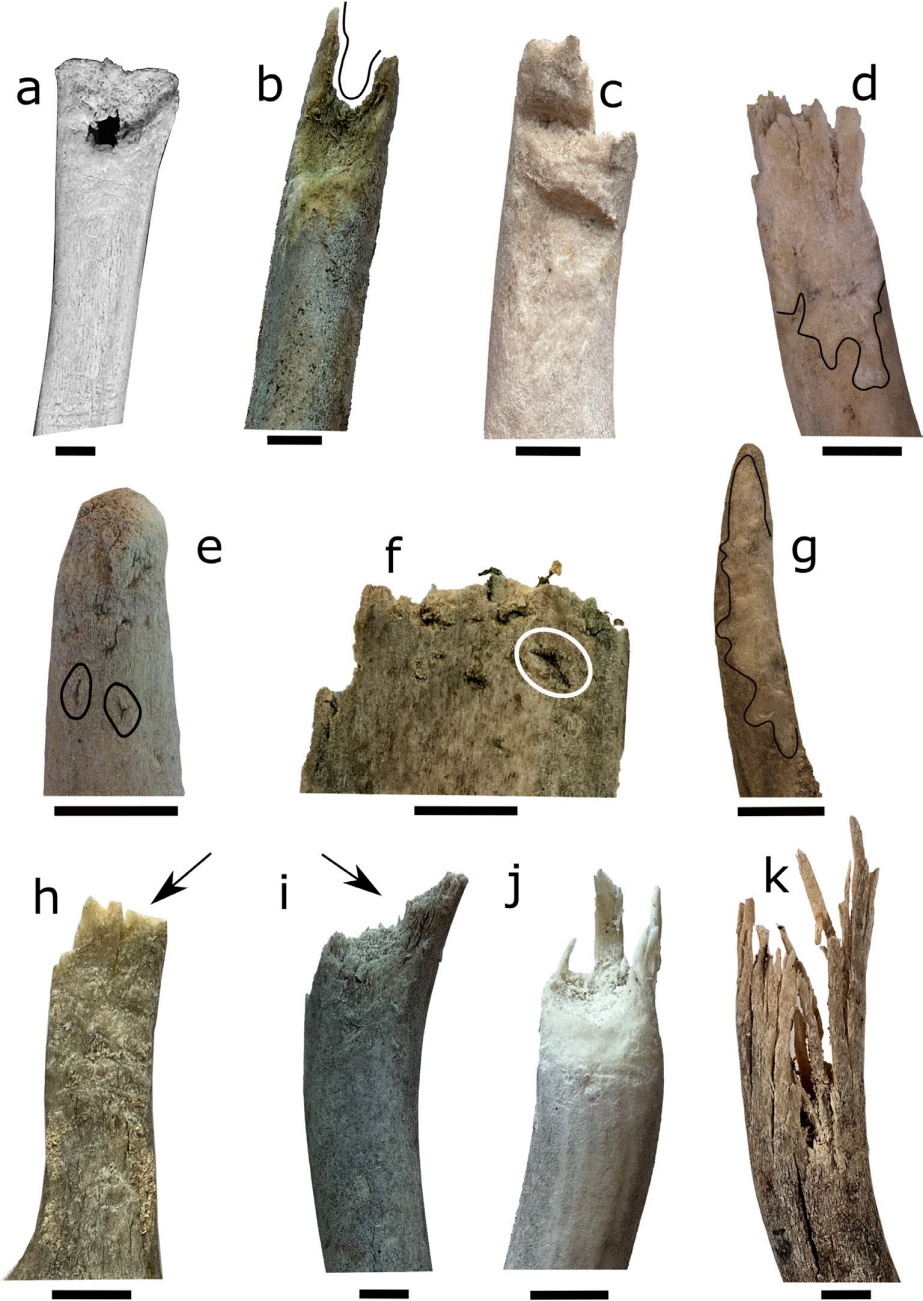
Ruminant modification classes, including those previously described (a–d) and novel classes described here and in the Atlas of Arctic Bone Modification (e–k; Gaetano et al. 2026). Modifications include (a) keyholes drilled into the distal end of a bone element described by Cáceres et al. 2011, but first identified by (Tokunaga 1936), (b) forked fractures described by (Sutcliffe 1973; Cáceres et al. 2011), (c) deep, U‐shaped furrows that reshape the profile of the bone described by (Sutcliffe 1973; Wika 1982), (d) collections of overlapping furrows, such that the outermost bone layers are removed described by (Brothwell 1976; Cáceres et al. 2011). Features described for the first time here and in the Atlas of Arctic Bone Modification (Gaetano et al. 2026) include (e) linear and (f) tetrahedral punctures confined to the outer cortical bone, (g) overlapping furrows with varying amounts of cortical bone removal, and (h) fractures with compressed, relatively straight edges, (i) fractures with compressed, angled edges, (j) fractures in which the inner cancellous bone has been removed and the remaining cortical bone “shell” splinters in long (several centimeters) shards along one edge, and (k) fractures in which the inner cancellous bone has been removed and the remaining cortical bone “shell” splinters in long (several centimeters) shards around the remaining bone. We do not observe keyhole punctures [a, photo from Cáceres et al. 2011, reproduced here with permission] in antlers and bones from the Arctic Refuge Coastal Plain. Specimen field and University of Alaska Museum Earth Sciences collection (UAMES) accession numbers: (b) T01‐55‐18 42, UAMES 54481; (c) T01‐37‐15 65, UAMES 54471; (d) T01‐37‐15 19, UAMES 54479; (e) T01‐42‐15 15, UAMES 54467; (f) T01‐44‐15 01, UAMES 54475; (g) T01‐43‐15 12, UAMES 54472; (h) T01‐02‐10 56, UAMES 54473; (i) T01‐45‐18 51, UAMES 54474; (j) T01‐37‐15 91, UAMES 54482; (k) T01‐30‐14 61, UAMES 54483. All scale bars are 1 cm.

An additional challenge to understanding how ungulates and other taxa differentially utilize bones is that bone modification by ungulates is often misidentified. In fact, several of what are now understood to be stereotypical features of ungulate modification (e.g., Figure 1a) were originally attributed to human tool use (Tokunaga 1936). Our limited understanding of how ungulates modify and consume bone also imposes fundamental restrictions on how we understand bone recycling at community scales. Similarly, past and ongoing failures to adequately consider the roles of ungulate modification on bones bias our understanding of species interactions recorded by paleontological and archeological datasets. Here, we assess bone modifications left on an assemblage of bones in an extant Arctic ecosystem to evaluate the partitioning of this resource among co‐occurring ungulates, carnivorans, and rodents. Through this work, we expand the number and diversity of bone modification classes that are attributable to ungulates (e.g., caribou; *Rangifer tarandus* ) and highlight the nutritional significance of shed female antlers as an important mineral resource for parturient (calf‐bearing) migratory caribou. Our findings also indicate that osteophagy by nursing caribou may help explain the remarkably synchronous timing between when migratory female caribou shed their antlers and the birth of their young (Espmark 1971). Further, benefits to calf survival and maternal health provided by transporting and subsequently consuming limiting nutrients via antlers may be an unrecognized evolutionary driver that transferred this primarily male secondary sexual trait onto female caribou.

## 2 Ruminant Bone Gnawing

The majority of bone gnawing and/or consumption by ungulates has been observed within Ruminantia (foregut‐fermenting even‐toed hoofed mammals; artiodactyls), with documented examples by most extant groups: Cervidae, Antilocapridae, Bovidae, Giraffidae, and Moschidae (Sutcliffe 1973; Brothwell 1976; Prothero and Foss 2007; Cáceres et al. 2011; Monson and Hlusko 2018). Bone is also consumed by nonruminating artiodactyls (suids) and those considered pseudo‐ruminants (hippopotamids and camelids; Johnson and Haynes 1985; Greenfield 1988; Cáceres et al. 2013; Dudley et al. 2016). To our knowledge there is little, if any, evidence of bone gnawing by perissodactyls, which include Rhinocerotidae, Tapiridae, and Equidae (Cáceres et al. 2013). While this taxonomic bias in bone gnawing among ungulates is not commonly identified in the literature, because most taphonomic evaluations focus on ruminant bone gnawing (Sutcliffe 1973; Wika 1982; Cáceres et al. 2011; Hutson et al. 2013), we will refer to “ruminant” rather than “ungulate” modification and gnawing from this point forward.

The features and marks left on bones during modification are similar among ruminant species and visually distinct from those produced by other taxa, which is consistent with tooth morphologies and chewing mechanics shared by ruminants (Sutcliffe 1973; Hutson et al. 2013). Ruminants lack upper (premaxillary) incisors and share selenodont cheek teeth, which are marked by sharp crescent‐shaped grinding surfaces. This is distinct from the more rounded bunodont teeth of suids and ursids, the high‐crowned hypsodont teeth of equids, and sharp carnassials of felids and canids (Popowics and Fortelius 1997; Ungar 2010; Damuth and Janis 2011).

To gnaw on bones, ruminants hold them between their cheek teeth in a “cigar‐like” manner, grinding the bone between their upper and lower premolars and molars (Sutcliffe 1973). This chewing style has been observed in wild and captive populations (Sutcliffe 1973; Cáceres et al. 2013; Hutson et al. 2013). Grinding bone material in this manner can transform the gross morphology of bones into a distinctive “forked” shape, as central portions of the bone are preferentially broken away within the mouth and consumed (Figure 1b; Sutcliffe 1973). Ruminant gnawing can also develop deep (Figure 1c) and shallow (Figure 1d) furrowing, which may occur in tandem with the forked modification. These furrows are another distinctive characteristic of bones gnawed on by ruminants, having been extensively documented in populations of reindeer and wild caribou ( *Rangifer tarandus* spp.), as well as sheep, giraffe, fallow and red deer ( *Ovis aries* , *Giraffa camelopardalis* , *Dama dama* , *Cervus elaphus* ; Sutcliffe 1973; Brothwell 1976; Wika 1982; Cáceres et al. 2011; Hutson et al. 2013). Following more protracted ruminant gnawing, a keyhole opening can be drilled at the end and midline of a bone (Figure 1a; Cáceres et al. 2011). These features are distinctly ruminant in origin, but all of them develop after relatively extensive gnawing. This raises the question: what do bones look like after being only lightly modified by ruminants? Until we can identify the full spectrum of bone modifications made by ruminants, the roles and importance of bone nutrients in ruminant ecology will be underappreciated, as will the roles that ruminants play in terrestrial bone recycling processes.

## 3 Caribou Calving Grounds: A Natural Experiment in the Competition for Bone Minerals

Migratory caribou journey to the same region each year to give birth to their young (Skoog 1968; Griffith et al. 2002; Cameron et al. 2020). These calving grounds are an ideal study system for assessing patterns of bone modification and bone mineral resource partitioning among co‐occurring taxa and trophic groups (ruminants, rodents, and carnivorans). This is true, in part, because the landscape surfaces of caribou calving grounds are rich in the bones of caribou calves and adults that do not survive parturition or die during migration (Griffith et al. 2002; Miller et al. 2013). Additionally, calving grounds are rich in the shed antlers of female caribou (Miller et al. 2013). Caribou are the only extant cervid in which females annually grow antlers, and migratory populations are known to shed them within days of giving birth to their young (Espmark 1971; Reimers 1993). Hundreds of thousands of caribou may return to a single calving ground each year (Griffith et al. 2002; Haskell and Ballard 2007; Prichard et al. 2020), producing dense accumulations of shed antlers (10^2^–10^3^ antlers/km^2^; Miller et al. 2013). These antlers, which are composed of bone, offer copious mineral (e.g., calcium, phosphorus) resources for potential mammalian consumers. Additionally, slow Arctic decomposition and sedimentation rates allow antlers and bones to persist for centuries to millennia, increasing the accessibility of bone resources and establishing a system in which bone loss is driven by consumption rather than weathering and burial (Miller et al. 2021; Miller and Simpson 2022; Miller et al. 2023).

In addition to the ready availability of bones on caribou calving grounds, unique nutritional demands in Arctic settings make them a valuable study system for evaluating bone modification of caribou and co‐occurring taxa. For local carnivorans (e.g., *Ursus arctos, Canis lupus*), particularly those that reside permanently on the calving grounds ( *Gulo gulo* , *Vulpes lagopus* ), the spring calving season represents an important influx of prey and scavenging opportunities (Griffith et al. 2002). For resident rodents (e.g., *Dicrostonyx groenlandicus* , *Lemmus trimucronatus* , *Microtus* spp., *Urocitellus parryii* ) the seasonal influx of bones and antlers may provide a source of nutrition and/or an abrasive for wearing down their incisors (Klippel and Synstelien 2007). For migratory caribou, calving grounds are an endpoint of some of the longest terrestrial migrations on the planet (Joly et al. 2019), which impose their own set of nutritional and energetic demands (Boertje 1985; Duquette and Klein 1987).

Following spring calving, female caribou experience the confluent stresses of lactation, continued migration, and antler regrowth, which starts only a few weeks after shedding their annually grown antlers (Reimers 1993; Cameron et al. 2005; Oster et al. 2018). The concentration of calcium and phosphorus available in spring and summer forage can be at least temporarily insufficient to meet the high mineralogical demands of lactating females during this period (Oster et al. 2018). Antlers are rich in calcium and phosphorus, both of which are required to produce milk, develop new antlers, and reconstitute muscle and bone mass lost during migration (Wika 1982; Pis et al. 1994; Tajchman et al. 2020). Further, because antlers are disconnected from the circulatory system and exposed to the elements for months prior to shedding (Lincoln and Tyler 1999; Kierdorf and Kierdorf 2011), they may be in a similar state to the weathered bones that ruminants preferentially target for consumption (Sutcliffe 1973; Cáceres et al. 2011).

## 4 Methods

Antlers and bones evaluated here were collected from the Coastal Plain of the Arctic National Wildlife Refuge (Alaska), which is an important calving ground for the Porcupine Caribou Herd (Griffith et al. 2002, 2019; Caikoski 2020). These collections come from a larger effort to study Arctic bone accumulations and historical patterns of caribou migration, seasonal landscape use, and calving ground geography (Miller et al. 2013, 2021; Miller and Simpson 2022). Antlers were collected using 58 standardized bone surveys across the Coastal Plain of the Arctic Refuge between 2010 and 2018 (Miller et al. 2013, 2021, 2023). We also include antlers and bones that were encountered serendipitously on the landscape. While sampling occurred across nearly a decade, most specimens come from decades (and even centuries) prior to our fieldwork (Miller et al. 2021, 2023). This extended temporal history (and its associated multi‐generational biological insight) is possible due to the slow rates of bone weathering in Arctic settings (Miller and Simpson 2022). Antlers were the main focus of the study and all antlers encountered during the surveys were collected. Approximately half of the skeletal bones were also collected, while the rest were left in the field due to logistical constraints. Skeletal bones were collected without reference to modification status and should be reasonably representative of the source population. Collection efforts produced 1567 shed caribou antlers and 224 adult and subadult skeletal bones of caribou, moose ( *Alces alces* ), and muskox ( *Ovibos moschatus* ). Bones of caribou calves were also recovered, but will be evaluated elsewhere because their high fragility subjects them to different taphonomic pathways than the more mineralized bones of older individuals (Faith and Behrensmeyer 2006; Miller et al. 2013).

To identify the modifying agents associated with each bone modification, we first conducted an extensive literature review, compiling previously identified modification features generated by taxa present on the Coastal Plain (Sutcliffe 1973; Brothwell 1976; Wika 1982; Haynes 1983; Barrette 1985; Klippel and Synstelien 2007; Pobiner et al. 2007; Pobiner 2008; Reeves 2009; Cáceres et al. 2011; Fosse et al. 2012; Hutson et al. 2013; Fernández‐Jalvo and Andrews 2016; Pokines et al. 2017; Arilla et al. 2019; Rosell et al. 2019; Eller et al. 2020; Keyes et al. 2020; Rassadnikov 2021; Pokines 2021; Gaetano et al. 2026). This review yielded attributable features of bone modification for each species, as well as more generalizable features of modification generated by each taxonomic group. We also compiled data on bone modifications that are not associated with tooth and jaw movements, including trampling and damage accrued by antlers while still attached to the individual (Haynes and Stanford 1984; Olsen 1989; Jin and Shipman 2010; Courtenay et al. 2020). To identify the suite of modifications made on skeletal bones and antlers from the Arctic Refuge's Coastal Plain, we then visually evaluated several hundred antlers and skeletal bones spanning the spectrum of modification features, gnawing intensities, and bone weathering conditions. We identified each class of bone modification present within the test antler set, describing them in detail and categorizing them as pits, punctures, scores, furrows, or fractures (Pobiner 2008; Fernández‐Jalvo and Andrews 2016). Pits and punctures are depressions in bones that typically conform to the tooth shape of the modifying agent (Figure A4; Pobiner 2008). Pits are shallow depressions that are confined to outer cortical bone, while punctures are deeper and extend into more internal bone layers (Figure A5; Pobiner 2008). The pits and punctures made by mammalian carnivores are perhaps the most frequently described and are generally circular in map view, reflecting the conical cusps of carnivoran cheek teeth (Pobiner et al. 2007; Fosse et al. 2012; Haynes and Hutson 2020). Scores and furrows are linear features, often with U‐shaped cross‐sections, that have lengths that are larger than their breadths. When resulting from a biological agent, these features are generally derived from a tooth, claw, or beak being dragged across a bone surface (Figures A6 and A7; Reeves 2009; Keyes et al. 2020). Scores and furrows may also form when bones interact with the environment, including when stepped on by a large mammal, ground across rocks and sediment, or rubbed against trees (in the case of antlers; Haynes and Stanford 1984; Olsen 1989; Ramos et al. 2006; Jin and Shipman 2010; Courtenay et al. 2020). Scores are restricted to the outer layers of bone. Furrows are deeper, generally larger, and penetrate into the cancellous bone that underlies cortical bone (Figure A8; Pobiner 2008). Fractures are broken bone margins and can have a variety of morphologies (straight, irregular, conchoidal) depending on the modifier, the underlying bone structure, and the moisture content of the bone (Pobiner et al. 2007; Karr and Outram 2012). After cataloging every unique pit, puncture, furrow, score, and fracture, each feature class was assigned an alphanumeric code (letters to indicate modification categories and numbers to separate class types within those categories; see Figure A3 and Table A1; Gaetano et al. 2026). During the process of defining modification classes, both MG and JM had to agree that a new candidate class was sufficiently distinct that it could be clearly defined and recognizable by the broader community. Detailed descriptions of observed modification classes and explanations of their attributed modifiers are provided within the Atlas of Arctic Bone Modification (Gaetano et al. 2026).

After documenting the diversity of bone modification classes observed on collected antlers and bones, we then assigned each class to a likely agent of modification using previously identified features that are diagnostic of particular species or taxonomic groups (Sutcliffe 1973; Wika 1982; Klippel and Synstelien 2007; Reeves 2009; Cáceres et al. 2011; Fosse et al. 2012; Fernández‐Jalvo and Andrews 2016; Arilla et al. 2019; Rosell et al. 2019; Rassadnikov 2021; Gaetano et al. 2026). Because of the challenges in attributing bone modifications to particular species, we followed others in attributing bone modification classes to broad taxonomic groups: carnivoran, rodent, and ruminant (Domínguez‐Rodrigo and Piqueras 2003; Pobiner 2008). For some modification classes, a modifying agent could not be confidently assigned or, in the case of antlers, could be attributed to caribou behaviors that scrape, fracture, and otherwise damage antlers while they are still attached (Olsen 1989; Jin and Shipman 2010).

On the Porcupine Caribou calving grounds, likely bone modifiers include carnivorans ( *Ursus arctos* , *Canis lupus* , *Vulpes vulpes* , *Gulo gulo* ), rodents ( *Dicrostonyx groenlandicus* , *Lemmus trimucronatus* , *Microtus* spp., *Urocitellus parryii* ), and ruminants ( *Rangifer tarandus* , *Ovibos moschatus* , *Alces alces* ; Babcock 1987; Cuyler et al. 2020; Lenart 2021; Johnson et al. 2022). Golden eagles ( *Aquila chrysaetos* ) and other avian raptors are common on the Coastal Plain and actively hunt or scavenge caribou calves (Griffith et al. 2002), though we found no features consistent with avian modification on any of the antlers or adult/sub‐adult skeletal bones. Caribou are the most prevalent ruminant in the area; the Porcupine Caribou Herd alone has over 200,000 individuals (Russell and Gunn 2019; Caikoski 2020). While muskox are also present on the Coastal Plain of the Arctic Refuge, they are orders of magnitude less abundant than caribou, having been locally extirpated in the early 1900s, reintroduced in 1969, and suffered major population declines in the early 2000s (Arthur and Del Vecchio 2017). Currently, there are likely several hundred muskox in the study region (Arthur and Del Vecchio 2017; Lenart 2021). While muskox are year‐round residents of the Coastal Plain (Cuyler et al. 2020), the area is covered in snow for the majority of the year (Sturtevant et al. 2012) and bone gnawing is likely biased to spring and summer months, when bones on the landscape are exposed, demands for phosphorus and calcium are highest, and caribou are the most numerous locally (Griffith et al. 2002; Oster et al. 2018). Additionally, while the geographic range of Alaskan moose ( *Alces alces* ) has extended north in recent decades, they generally live south of our Coastal Plain sampling area (Tape et al. 2016). Thus, it is likely that all or nearly all observed ruminant modifications are due to caribou activity.

**FIGURE A1:**
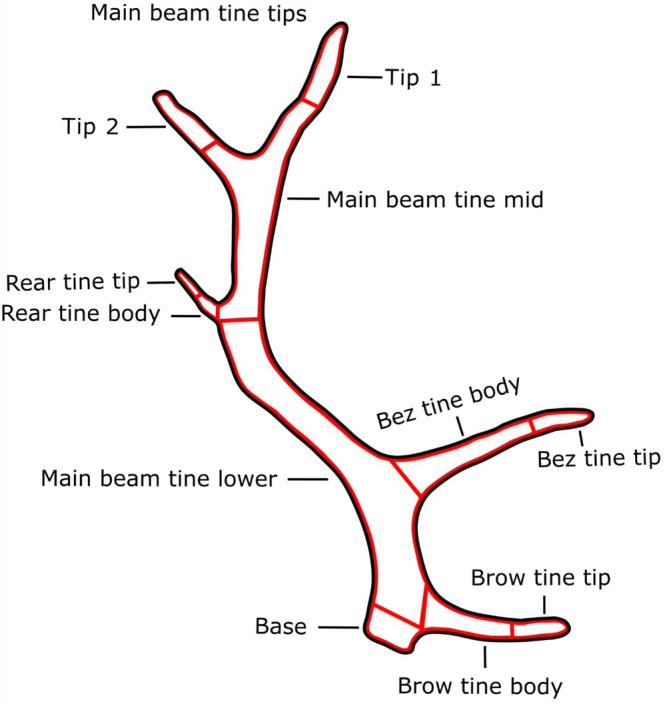
Anatomical landmarks used to delineate the location of individual modification features on an antler. When tines had multiple tips, each one was identified in the manner of the example shown here for the two tips of the main beam; figure from (Gaetano et al. 2026).

**FIGURE A2:**
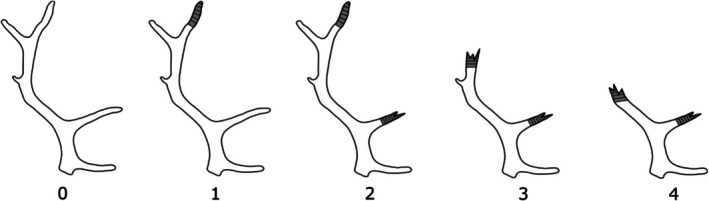
Ruminant Gnawing Intensity (GI) scale. GI 0 indicates an antler that was not modified by a ruminant. GI 1 is an antler with gnawing present, but no tines removed by gnawing. GI 2 indicates an antler with a single tine removed by ruminant gnawing. GI 3 is an antler with 2 or more tines removed by ruminant gnawing. GI 4 is an antler with the main beam removed below (proximal to) the rear tine.

To catalog taphonomic modifications on all collected antlers and skeletal bones, MG visually inspected each specimen and documented the class and location of all modifications (or lack thereof). Developing standardized procedures for locating modifications on caribou antlers is complex because they are morphologically variable in the number of points (terminating ends) and tines (individual branches) that emanate from the main antler beam (central and longest branch), the degree of palmation (flattening out) along the antler, as well as the presence and absence of different tines (e.g., many but not all antlers have separate brow and bez tines near the base of the antler, Figure A1; Miller 1986). To map modifications on a standardized antler “map”, we divided antlers into 10 regions, defined by five anatomical landmarks (see Appendix, Figure A1). For antlers, we also developed a semi‐quantitative scale of ruminant gnawing intensity (GI) ranging from 0 (no ruminant modification) to 4 (ruminant‐attributed removal of multiple regions of antler anatomy; Appendix: Methods and Figure A2). We restricted this analysis to modifications generated by ruminant (caribou) gnawing, which are the dominant modifiers of antlers in this region (see Results: Section 5).

Analytically, we used a chi‐square test (and the standardized residuals of that model) to evaluate potential differences in the frequencies with which antlers and skeletal bones were gnawed by caribou, carnivorans, and rodents. Following standard protocols, standardized residuals with magnitudes greater than 3.0 (positive or negative) are considered strongly distinct (Agresti 2007). To quantify potential differences with which different anatomical portions of antlers are gnawed on by caribou, we used a null model approach to establish the expected frequency with which any given antler portion should be gnawed, based on the structure and sampling of our data. To do this, we used a permutation approach to randomly shuffle the assignments of both the antler portion and whether that portion was gnawed or not (Gotelli 2001; Gotelli and McGill 2006). This was repeated 10,000 times to establish the expectation that any given antler portion should be gnawed. We then compared this null expectation (expressed as a 95% confidence interval) to our empirical values to test whether each antler portion had higher or lower frequencies of gnawing. We paired this analysis with binomial tests (gnawed vs. not‐gnawed) for each antler portion. All analyses were conducted in the open‐sourced statistical framework, R (version 4.4.3; R Core Team 2024).

## 5 Results

### 5.1 Modification Classes

**FIGURE 2:**
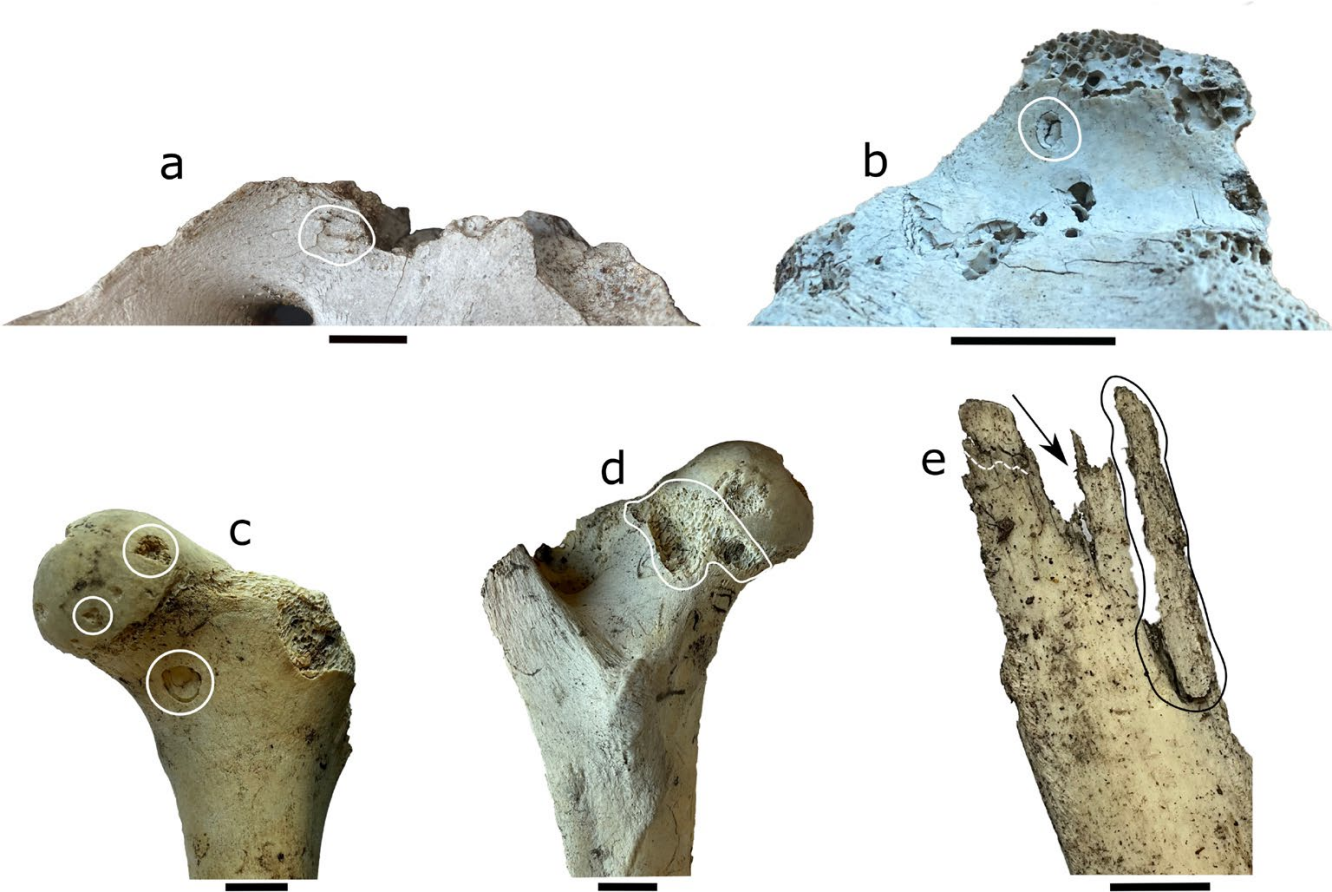
Classes of bone modification generated by Arctic carnivorans. These include (a, b) pits (Sacral vertebrae of a moose, T01‐28‐14 14, UAMES 54477 and thoracic vertebra of a caribou, T01‐20‐14 20, UAMES 54476), (c) punctures (caribou femur: T01‐19‐14 30, UAMES 54469), (d) furrows (caribou femur: T01‐19‐14 30, UAMES 54469), and (e) fractures (caribou rib, T01‐14‐12 03, UAMES 54489). See Appendix for additional information on each specimen. Scale bars are 1 cm.

**FIGURE 3:**
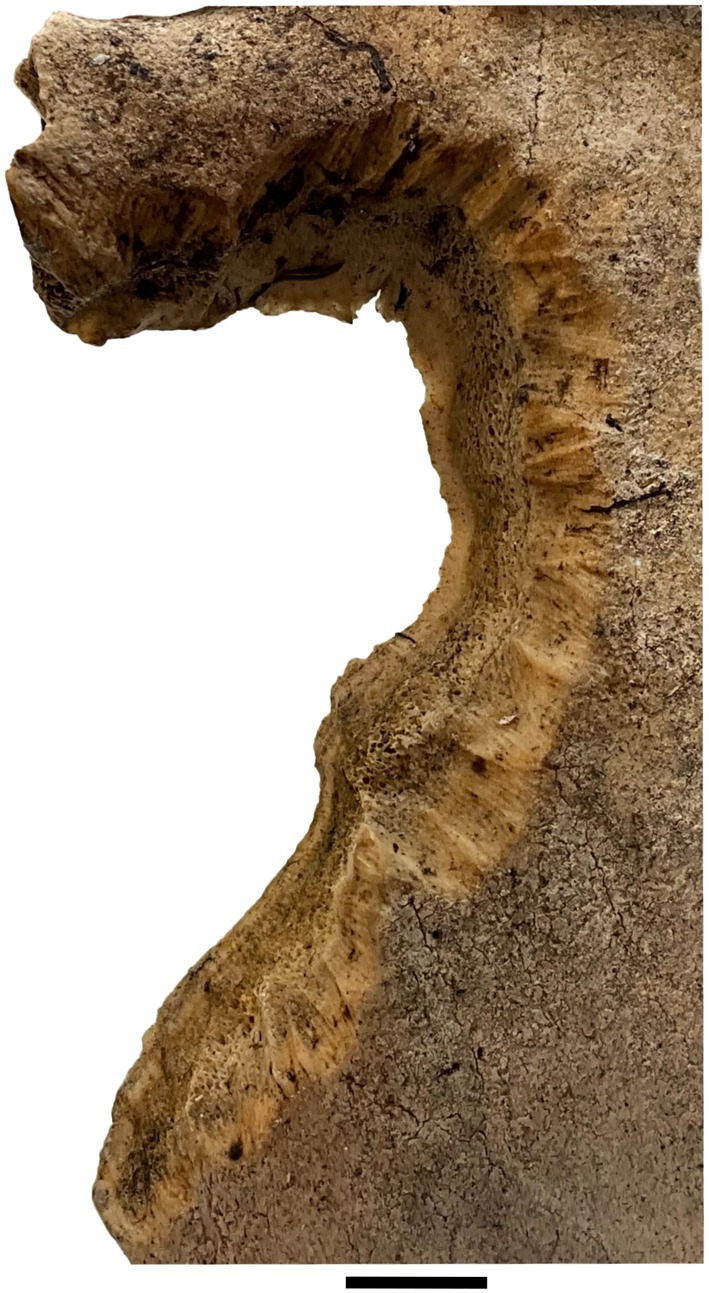
Parallel furrows on a caribou antler generated by rodent gnawing (specimen T05‐03‐22 02, UAMES 54480). Scale bar is 1 cm.

**FIGURE A3:**
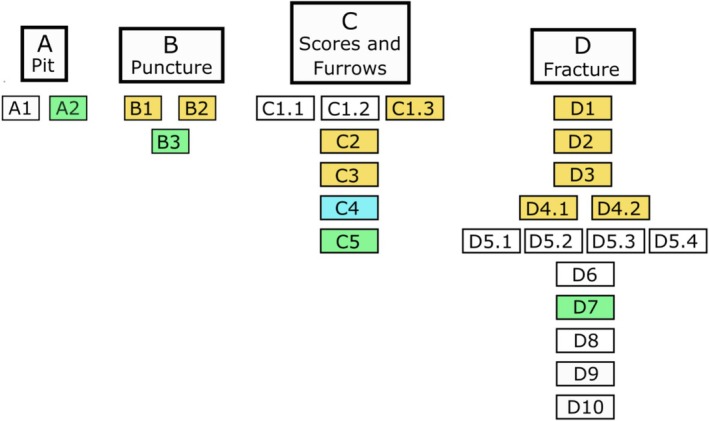
Alphanumeric codes for modification classes identified on antlers and skeletal bones from the Coastal Plain of the Arctic Refuge. The modifying agent is color‐coded: Carnivoran in green, ruminant (caribou) in yellow, rodent in blue, unattributed in white.

**FIGURE A4:**
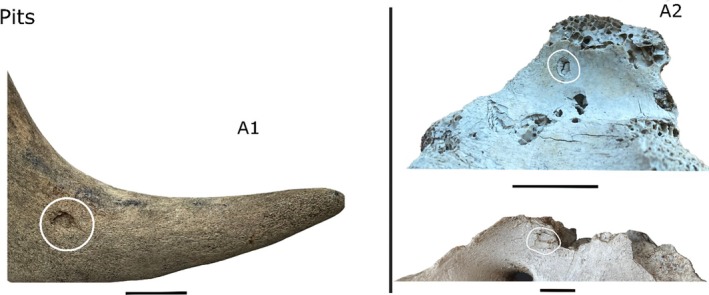
Example images highlighting pit classes A1 (left) and A2 (right) observed on antlers and/or skeletal bones from the Coastal Plain of the Arctic National Wildlife Refuge. Photographed specimens are identified by both field collection numbers, which begin with “T0” or “ANWR” and University of Alaska Museum Earth Sciences (UAMES) accession numbers. Specimens photographed here include caribou antler T01‐42‐15 15, UAMES 54467 (A1), a thoracic vertebra of a caribou, T01‐20‐14 20, UAMES 54476 (A2 bottom), and a sacral vertebrae of a moose ( *Alces alces* ), T01‐28‐14 14, UAMES 54477 (A2 top). White outlines on bones highlight modification features in their respective images. See text, Table A1, and The Atlas of Arctic Bone Modification (Gaetano et al. 2026) for descriptions of individual classes and the agent responsible for that modification class. Image modified from (Gaetano et al. 2026). Scale bars are 1 cm.

**FIGURE A5:**
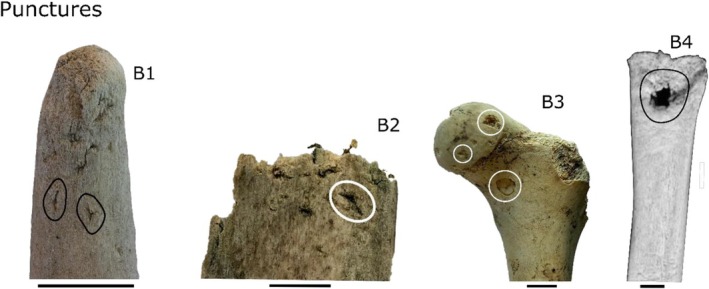
Example images highlighting different classes (B1, B2, B3, B4) of punctions observed on antlers and/or skeletal bones from the Coastal Plain of the Arctic National Wildlife Refuge and elsewhere. Specimens photographed include caribou antler T01‐42‐15 15, UAMES 54467 (B1), caribou antler T01‐44‐15 01, UAMES 54475 (B2), and a caribou femur, T01‐19‐14 30, UAMES 54469 (B3). Class B4 was not observed on the Coastal Plain, but is a known modification class produced by cervid species (Cáceres et al. 2011). Black and white circles on bones highlight modification features in their respective images. See text, Table A1, and The Atlas of Arctic Bone Modification (Gaetano et al. 2026) for descriptions of individual classes and the agent responsible for that modification class. Image modified from (Gaetano et al. 2026). Scale bars are 1 cm.

**FIGURE A6:**
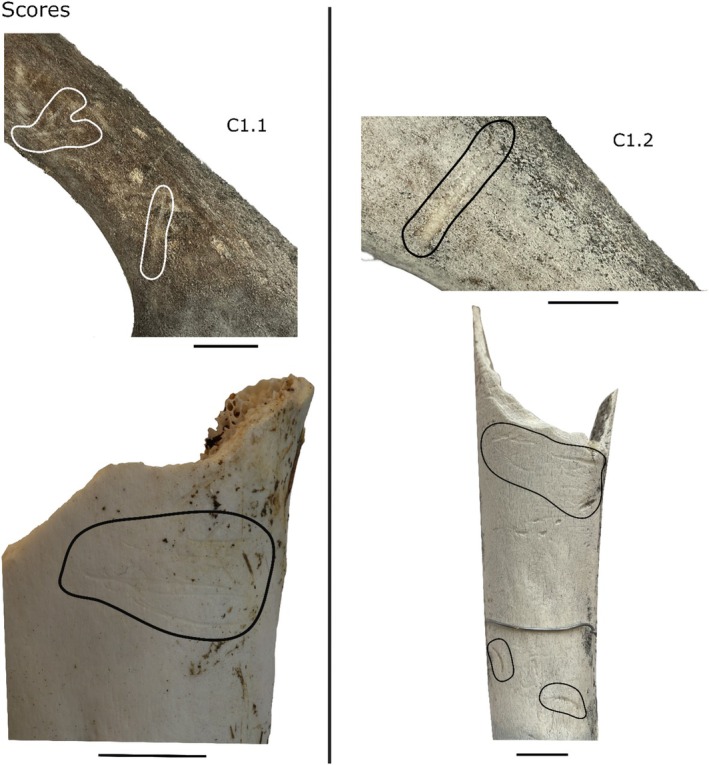
Example images highlighting different classes (C1.1 on left side; C1.2 on right side) of scores observed on antlers and/or skeletal bones from the Coastal Plain of the Arctic National Wildlife Refuge. Black and white outlines on bones highlight modification features in their respective images. Specimens photographed here include caribou antler T01‐04‐10 99A, UAMES 54468 (C1.1 top and C1.2 top), a femur of a caribou, T01‐55‐18 46, UAMES 54470 (C1.1 bottom), and a femur of a caribou, T01‐53‐18 52, UAMES 54478 (C1.2 bottom). See text, Table A1, and The Atlas of Arctic Bone Modification (Gaetano et al. 2026) for descriptions of individual classes and the agent responsible for that modification class. Image modified from (Gaetano et al. 2026). Scale bars are 1 cm.

**FIGURE A7:**
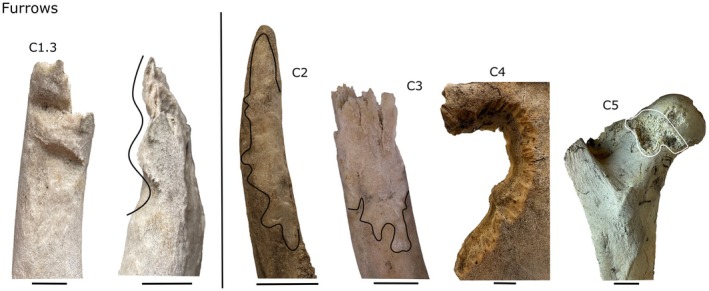
Example images highlighting different classes (C1.3, C2, C3, C4, C5) of furrows observed on antlers and/or skeletal bones from the Arctic National Wildlife Refuge. C2 and C5 furrows are outlined in their respective images. Specimens photographed here include caribou antler T01‐37‐15 65, UAMES 54471 (C1.3), antler T01‐43‐15 12, UAMES 54472 (C2), antler T01‐37‐15 19, UAMES 54479 (C3), antler T05‐03‐22 02, UAMES 54480 (C4), and a caribou femur, T01‐19‐14 30, UAMES 54469 (C5). See text, Table A1, and The Atlas of Arctic Bone Modification (Gaetano et al. 2026) for descriptions of individual classes and the agent responsible for that modification class. Image modified from (Gaetano et al. 2026). Scale bars are 1 cm.

**FIGURE A8:**
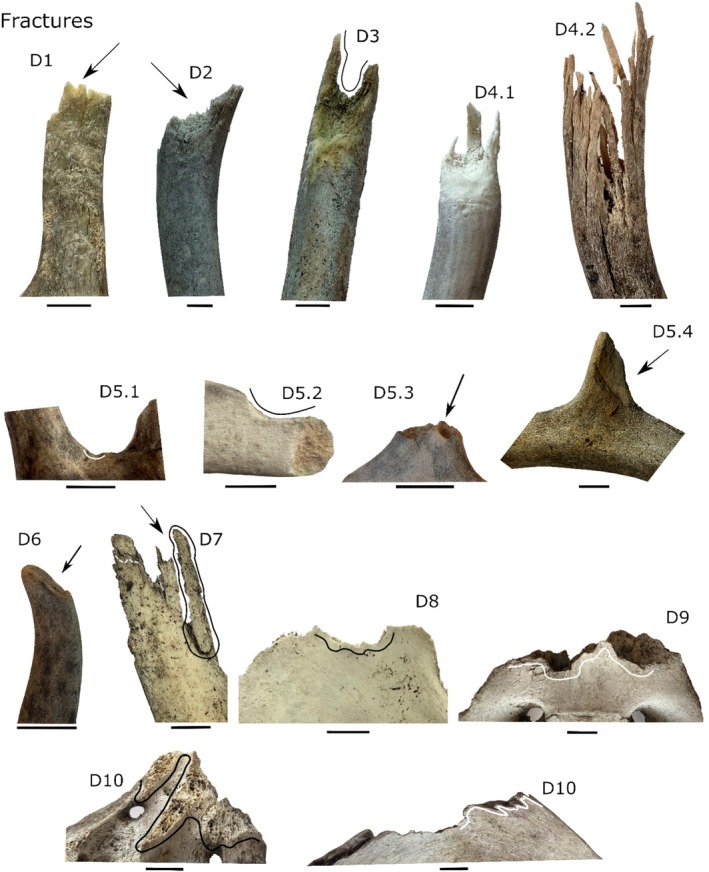
Example images highlighting different classes (D1, D2, D3, D4.1, D4.2, D5.1, D5.2, D5.3, D5.4, D6, D7, D8, D9, D10) of fractures observed on antlers and/or skeletal bones from the Coastal Plain of the Arctic National Wildlife Refuge. Arrows and outlines highlight modification features in their respective images. Photographed specimens include caribou antler T01‐02‐10 56, UAMES 54473 (D1), antler T01‐45‐18 51, UAMES 54474 (D2), antler T01‐55‐18 42, UAMES 54481 (D3), antler T01‐37‐15 91, UAMES 54482 (D4.1), antler T01‐30‐14 61, UAMES 54483 (D4.2), antler T01‐56‐18 50B, UAMES 54484 (D5.1), antler T01‐44‐15 05, UAMES 54485 (D5.2), antler T01‐43‐15 34, UAMES 54486 (D5.3), antler T01‐02‐10 22, UAMES 54487 (D5.4), antler T01‐58‐18,127, UAMES 54488 (D6), a caribou rib, T01‐14‐12 03, UAMES 54489 (D7), an innominate of a caribou calf, T01‐56‐18 20, UAMES 54490 (D8), a moose sacrum T01‐28‐14 14, UAMES 54477 (D9 and D10 left), and a caribou mandible ANWR 251, UAMES 54491 (D10 right). See text, Table A1, and The Atlas of Arctic Bone Modification (Gaetano et al. 2026) for descriptions of individual classes and the agent responsible for that modification class. Image modified from (Gaetano et al. 2026). Scale bars are 1 cm.

We documented 27 distinct modification classes on bones and antlers from the Arctic Coastal Plain. This includes two pits, four punctures, two scores, five furrows, and fourteen fractures (Figure A3, Table A1, see Appendix; Gaetano et al. 2026). Nine of these classes have previously been attributed to known modifiers: four to caribou (ruminant) modification (Figure 1b–d), four to carnivorans (Figure 2), and one to rodents (Figure 3; see Appendix for additional details; Gaetano et al. 2026). Keyhole fractures (B4; Figure 1a) were the only previously documented class of ruminant modification that we did not observe. We observed one pit, two scores, and eight fracture classes that were consistent with modifications that may have developed while the antlers were still attached to the head, or which were too generic in appearance to confidently attribute to a single modifying taxon (see Table A1; Olsen 1989; Jin and Shipman 2010; Gaetano et al. 2026). We attributed seven of the remaining modification classes to caribou gnawing based on shared features of previously described patterns of caribou and/or cervid modification and consistencies with the dental anatomy and chewing mechanics of caribou (see Appendix; Sutcliffe 1973; Wika 1982; Cáceres et al. 2011; Gaetano et al. 2026).

The new classes of modification included two puncture classes (one linear and one tetrahedral; Figure 1e,f), one class of chaotically overlapping furrows, which partially extend into cortical bone (Figure 1g), and four fracture classes (Figure 1h–k, see Appendix, Gaetano et al. 2026). Both puncture classes are visually distinct from circular and/or conical punctures generally associated with bone modification by carnivorans (bear, wolf; Pobiner et al. 2007; Rosell et al. 2019) and are instead consistent with the roughly tetrahedral cusps of caribou premolars and molars (see Appendix). The newly identified class of furrows (Figure 1g) is visually similar to previously described cervid modification, but distinguishable by their more limited removal of cortical bone (see Appendix; Cáceres et al. 2011; Gaetano et al. 2026). The four fracture classes attributed to caribou (Figure 1h–k) are also consistent with, and have high co‐occurrence (58%–95%) with previously described fractures and other bone modification features generated by caribou and other cervids (see Table A1; Sutcliffe 1973; Wika 1982; Cáceres et al. 2011; Hutson et al. 2013; Gaetano et al. 2026). Complete descriptions of all modification classes, including their co‐occurrence with ruminant modification classes, are available in the Appendix (Table A1) and in this paper's companion photographic atlas (Gaetano et al. 2026).

### 5.2 Modifications Observed on Antlers and Skeletal Bones

**FIGURE 4:**
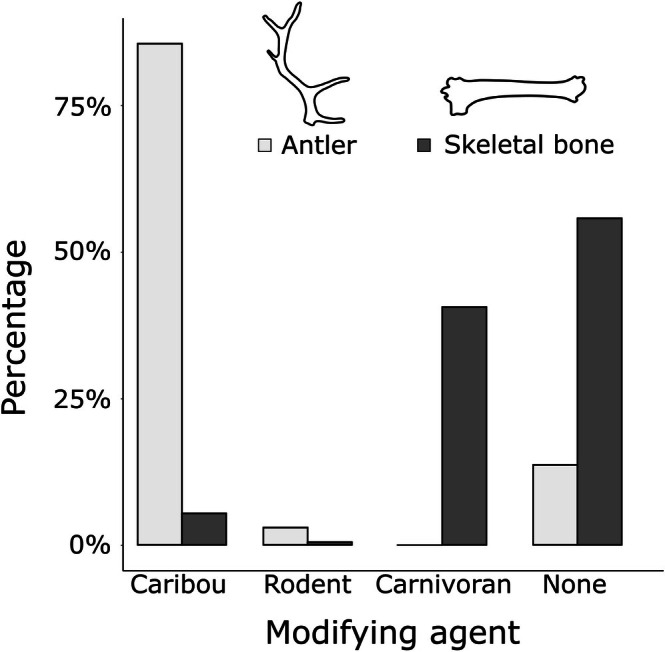
Percentages of antlers (light gray) and skeletal bones (dark gray) modified by caribou (ruminants), rodents, or carnivorans. The percentages of unmodified antlers and bones are also provided. Percentages reflect those in Table 1.

We documented 8724 occurrences of modification on shed antlers collected from the calving grounds, of which 6496 (74.5%; Table A1) are attributable to caribou gnawing. 3621 of these occurrences (41.5%) are of ruminant (caribou) modification classes that are identified here for the first time (see Figure 1 and Table A1; Gaetano et al. 2026). Overall, 1341 antlers (85.6%; Figure 4) have modifications attributable to caribou gnawing, with 239 of those (17.8%) only identified by the presence of one or more of the seven novel classes identified here (Figure 1e–k). Rodents were the only other observed modifier of antlers, but we found rodent modification (Figure 4) on only 48 (3.1%) antlers. We did not observe any carnivoran modifications on shed antlers.

Unlike shed antlers, the majority of skeletal bones were unmodified (125 of 224; 55.8%, Figure 4). Carnivorans were the most common modifier of skeletal materials (*n* = 91 bones, 40.6%). We observed 12 skeletal bones (5.4%) with caribou‐attributed modification, and one (0.4%) with rodent modifications. A chi‐square test (*X* ^2^ = 993.49, df = 3, *p* < 0.001) and associated standardized residuals (Table 2) confirm that differences in gnawing frequencies of antlers vs. skeletal bones between the three candidate bone consumers are strongly significant. Caribou (and to some extent rodents) clearly target shed antlers over available skeletal bones, while carnivorans were only observed to modify skeletal bones. Additionally, while the vast majority of antlers show some sign of gnawing, the mineral resources available in skeletal bones are comparatively underutilized (Figure 4 and Table 2).

**FIGURE 5:**
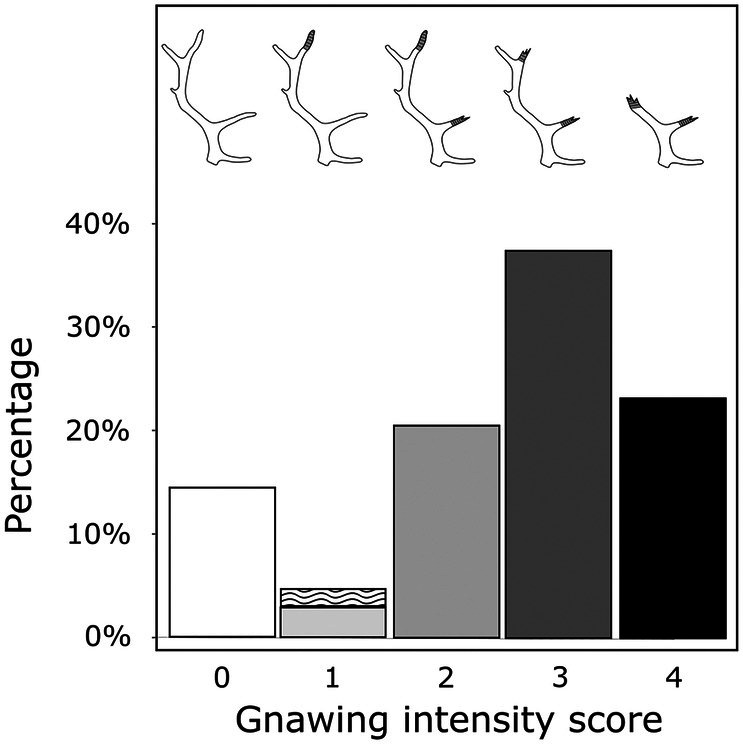
Percentage of shed antlers in each class of caribou gnawing intensity (GI). Graphical depictions of the five‐part intensity scale are shown above the data. Antlers in GI 1 are partitioned into those identified as caribou‐gnawed using the presence of linear and/or tetrahedral punctures first described here (solid portion of GI 1 bar) and those exhibiting one or more previously defined classes of ruminant modification (wavy portion of bar).

**FIGURE 6:**
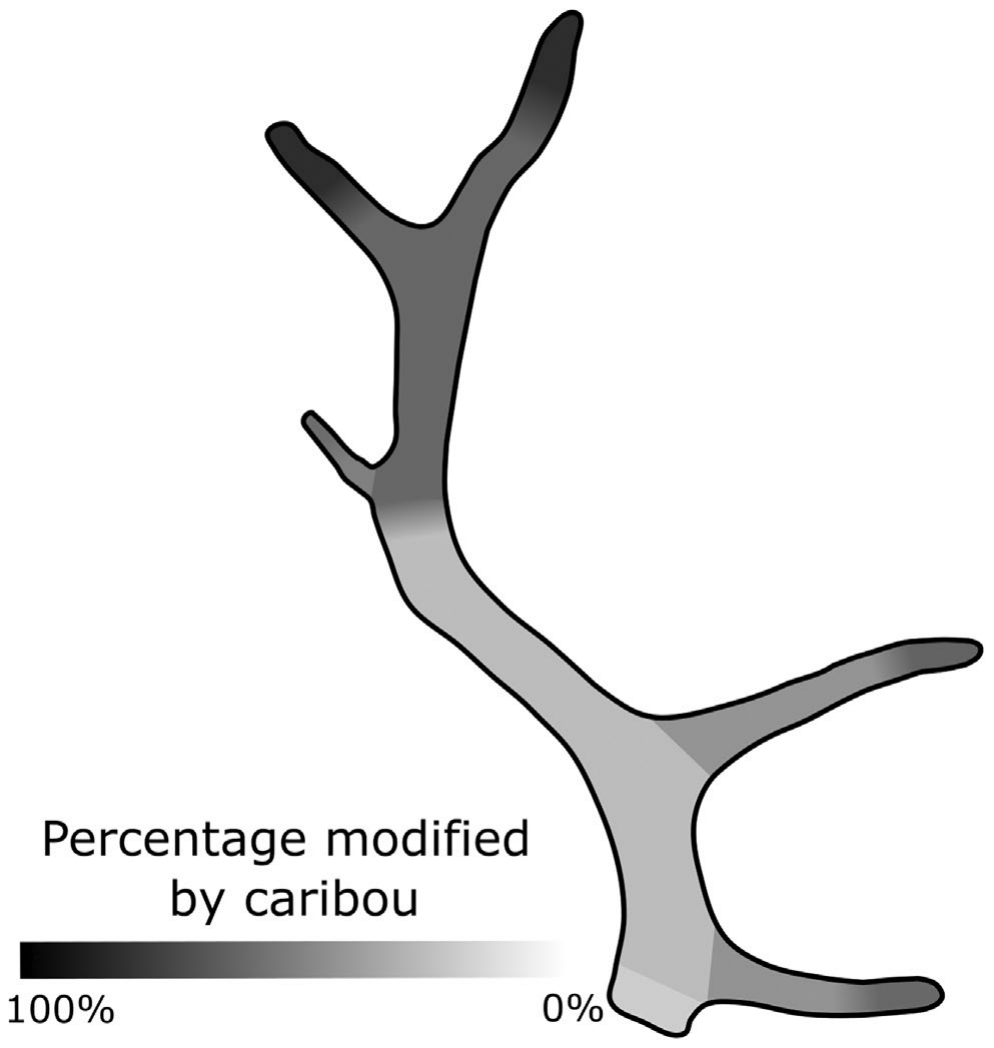
Schematic illustrating the percentage with which each of the ten antler regions is modified by caribou. Among all evaluated antlers, the majority (81%) record caribou modification on the distal tips of the main beam. Modification is also common along the outer, distal tips of the brow, bez, and rear tines, but less common along the bodies of each respective tine. Modification on antler bases is more rare (18% of antlers). Due to variability in antler form, we use a shading gradient between the tips and bodies of antler tines to indicate uncertainty in how regions of a particular antler are mapped onto this generalized schematic.

**FIGURE 7:**
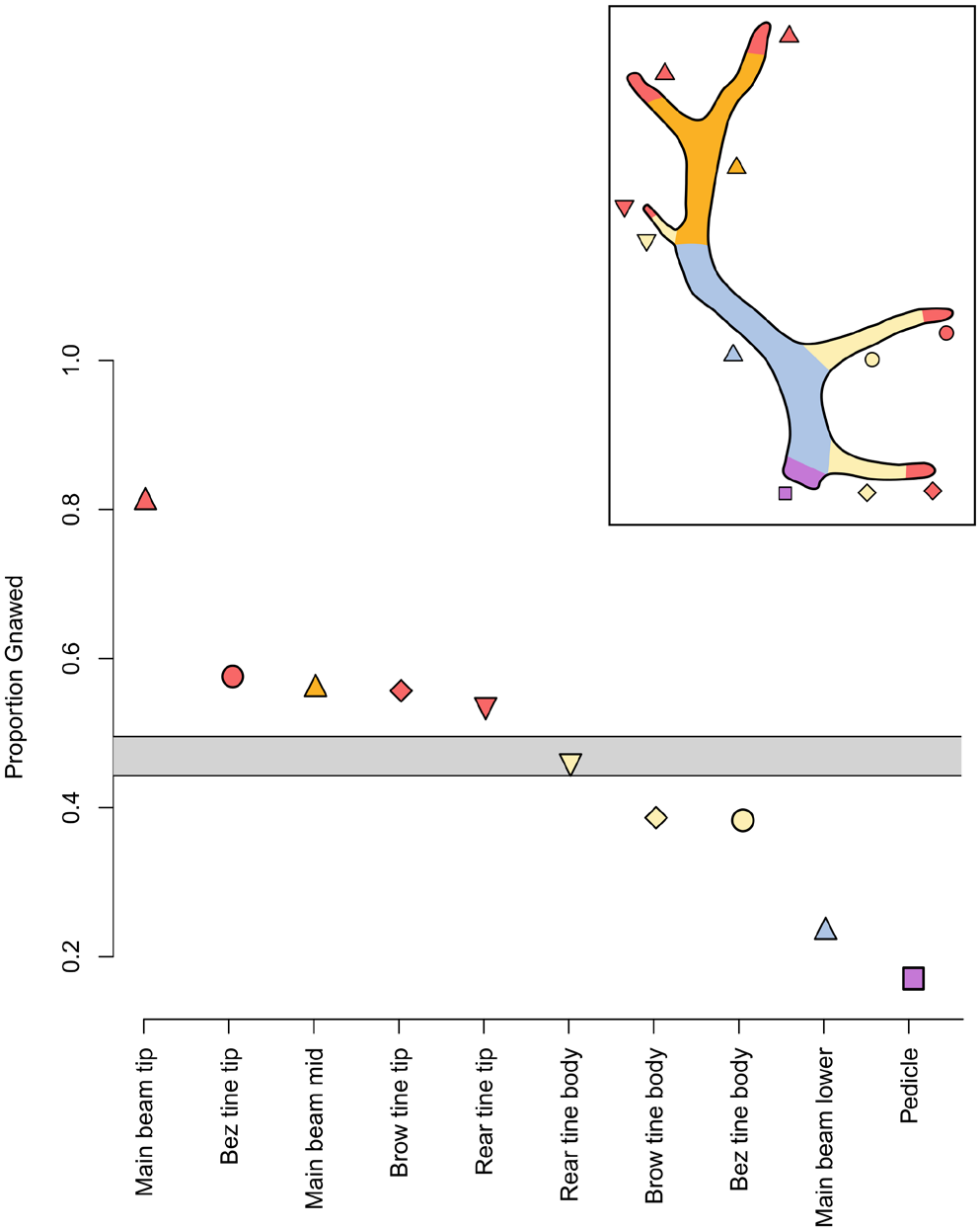
The proportion with which each of the 10 antler regions (horizontal axis; antler inset) are gnawed by caribou. Horizontal gray bar indicates the 95% confidence interval for the null expectation of gnawing, given our sampling. Points above the null expectation (tips of all antler tines and the upper main beam) indicate that gnawing is significantly more frequent than expected. Points below the null expectation (lower bodies of most tines, lower main beam, and pedicle) indicate that gnawing is significantly less frequent than expected. Shapes of points (see inset) correspond to antler region (e.g., bez tine vs. brow tine). Colors of points correspond to shared locations (e.g., tips of tines vs. lower portions of tines).

Focusing on the intensity with which antlers are gnawed by caribou, we found that the majority are heavily or moderately modified (23.2% and 37.4% in GI 4 and GI 3, respectively; Figure 5). Far fewer are either lightly modified (20.5% and 4.5% in GI 2 and GI 1, respectively) or unmodified by caribou (14.4% in GI 0; Figure 5). We also find clear directionality in the progression of caribou gnawing on antlers; antlers are gnawed from the distal tips towards antler bases (Figure 6). This is confirmed by our null model (Figure 7) and binomial tests (all *p* < 0.001 except for the base of the rear tine [*p* > 0.05]), which indicate that all tine tips (and the upper main beam) are significantly more gnawed than expected. Concurrently, we found that tine bases (with the exception of the rear tine), the lower portion of the main beam, and the pedicle are significantly less gnawed than expected. While gnawing near antler bases does occur, it is rare (278 antlers, 17.7%), relative to the frequency with which distal tines of the main beam are gnawed (1,277 antlers, 81.5%).

Directionality of antler consumption by rodents is challenging to evaluate, given that we documented only 78 occurrences of rodent gnawing (on 48 antlers, 77 of these occurrences could be confidently assigned to specific antler regions). However, most antler portions (main beam, brow tine, bez tine, rear tine) record greater frequencies of rodent gnawing on their distal tips (48 occurrences, 62.3%) relative to the main body of those regions (18 occurrences, 23.4%) or antler pedicles (11 occurrences, 14.3%). These differences are significant (*X* ^2^ = 30.1, df = 2, *p* < 0.001), once again illustrating excess gnawing on antler tips compared to antler pedicles (though with more equivocal results for the main bodies of individual antler tines; Table 3).

## 6 Discussion and Conclusions

Stark differences in tooth shapes, chewing mechanics, and known patterns of bone modification among ruminants, rodents, and carnivorans provide a strong foundation for differentiating damage among modifiers and identifying new modification classes made by ruminants (caribou; Sutcliffe 1973; Wika 1982; Klippel and Synstelien 2007; Cáceres et al. 2011; Hutson et al. 2013; Fernández‐Jalvo and Andrews 2016). For example, although ruminants and rodents both produce clusters of furrows and scores, features produced by rodents generally run parallel to one another or radiate out from a focal point (Klippel and Synstelien 2007; Fernández‐Jalvo and Andrews 2016; Pokines et al. 2017). Furrows generated by ruminants, on the other hand, are generally not as organized (Sutcliffe 1973; Wika 1982; Cáceres et al. 2011; Gaetano et al. 2026). Similarly, while both ruminants and carnivorans produce punctures, carnivoran punctures are generally conical and inconsistent with the shape of ruminant teeth (Sutcliffe 1973; Pobiner 2008; Fernández‐Jalvo and Andrews 2016). While the weight of evidence supports our attribution of ruminants (caribou) to the seven newly characterized modification classes (see Appendix; Gaetano et al. 2026), additional controlled feeding experiments with captive caribou and other ruminants will provide useful tests of our assessments.

Punctures produced by caribou (and likely ruminants more broadly) are visually distinctive and expand our capacity to identify ruminant‐modified bone. Forty‐five of the caribou‐modified antlers (63.4% of antlers in GI 1, solid portion of GI 1 in Figure 5, Table A1) were identifiable only by the presence of linear or tetrahedral punctures (Figure 1f). None of these would have been recognized as ruminant‐gnawed using previously established criteria. Linear and tetrahedral punctures can likely facilitate a more thorough catalog of ruminant bone modification in a variety of modern, historical, and fossil settings.

Our results indicate that caribou are prolific modifiers of shed antlers on the calving grounds of the Porcupine Caribou Herd. Not only do 85.6% of antlers show some indication of modification by caribou (Figure 4), 81% of antlers have a gnawing intensity score of 2 or higher, indicating sustained gnawing for long enough to remove portions of multiple tines (Figure 5). While we cannot differentiate between prolonged gnawing by a single individual versus serial consumption by multiple individuals over perhaps many years, it is clear that caribou routinely consume shed antler material. This finding aligns with past observations of female reindeer and caribou consuming antlers shed during the calving interval (Kelsall 1957; Wika 1982). Additionally, because females comprise the bulk of caribou on the calving grounds (Wika 1982; Russell et al. 1993; Griffith et al. 2002), gnawing observed in this study is likely dominated by females.

Caribou undergo some of the longest migrations of any terrestrial mammal, with females of the Porcupine Caribou Herd traveling over 4000 km annually (Fancy et al. 1989; Joly et al. 2019). During these migrations, females must accommodate a host of mineralogical stresses, including simultaneously nursing their young while growing a new set of antlers and replacing skeletal and muscular resources used to survive winter, travel daily, and grow their fetus (Moen and Pastor 1998; Oster et al. 2018). These compounding demands require abundant calcium and phosphorus (Moen and Pastor 1998). Lactation is especially costly in terms of female caribou energy and mineral budgets, with nursing females recording lower body mass and poorer body condition, relative to females who are not nursing or leading a calf (Cameron et al. 2005). During portions of the spring and summer, forage can be deficient in calcium and phosphorus relative to the demands of nursing caribou, exerting further nutritional stress (Oster et al. 2018). The prodigious gnawing by caribou on their own shed antlers suggests that these calcium and phosphorus‐rich resources (Pathak et al. 2001; Richardson et al. 2008) likely serve as an important postnatal supplement for nursing females, likely during both spring and summer months.

Antlers, like all bones, are a complex mixture of hard and soft tissues, composed of approximately 35%–45% protein, 22% calcium, and 11% phosphorus (Pathak et al. 2001; Richardson et al. 2008). While antlers and skeletal bones contain similar concentrations of calcium, differences in the calcium binding capacity of antlers make calcium from antlers more bioavailable compared to other (skeletal) bones. This is highlighted by red deer antlers, which have a calcium binding capacity (53.68% ± 1.80%) that is nearly 50% higher than skeletal bone (36.69% ± 0.56%). In lab mice, the enhanced calcium uptake afforded by increased calcium binding capacity of antlers was enough to effectively counteract the effects of osteoporosis (Wang et al. 2022). Outside the lab, this finding aligns with seasonal increases in bone gnawing by a variety of species (including red deer, Cape porcupine [ *Hystrix africaeaustralis* ], gray squirrels [ *Sciurus carolinensis* ]) during birthing intervals (Carlson 1940; Gambín et al. 2017; Mori et al. 2018). While more direct studies are important for evaluating the physiological and overall biological benefits of consuming shed antlers for caribou, this nutritional resource may be broadly important for reducing the simultaneous mineralogical demands of producing high‐quality milk, continuing to undergo long‐distance migrations, and growing a new set of antlers (Landete‐Castillejos et al. 2019).

Antler consumption by caribou is not uniform across the body of the antler and is strongly directional. Gnawing is clearly concentrated along the distal portions of antlers, progressing proximally towards the antler base (Figures 6 and 7, Table 2). While there are some examples of gnawing on antler bases (pedicles), this is most frequently observed on antlers that are more heavily modified. Of the 278 occurrences of caribou gnawing on antler bases, the majority (229, 82.4%) were found on the most intensively gnawed antlers (those in GI 4 and GI 3). Rodent gnawing also tends to concentrate along the distal ends of antler tines, often accumulating where cancellous bone is exposed along a fractured surface. However, while our overall findings are significant, the relative rarity of rodent modifications on antlers (78 occurrences on 48 antlers) makes it challenging to more fully explore the patterns of rodent gnawing across different antler surfaces.

Why is antler gnawing by caribou so directional? Several morphological and behavioral factors likely contribute to this pattern. This includes the “cigar‐like” gnawing strategy used by ruminants (Sutcliffe 1973), which necessitates that modification accrues along the outermost bone regions (e.g., distal tines) prior to reaching more central regions. Differences in bone structure likely also play important roles. Antler tips are largely composed of lightly built and spongy cancellous bone that is covered by only a thin veneer of dense cortical bone. This changes dramatically towards the base of the antler, which is primarily composed of dense cortical bone (Landete‐Castillejos et al. 2007; Kierdorf and Kierdorf 2011; Landete‐Castillejos et al. 2019). By focusing gnawing activity on antler tips (Figures 6 and 7, Table 2), caribou are targeting regions of lower bone density that are likely more efficiently consumed. Importantly, this pattern does not reflect overall mineral availability, which is highest in denser bone (Chen et al. 2009). This suggests a trade‐off in favor of the ease of consuming cancellous‐rich bone over harder‐to‐acquire, but mineralogically richer, cortical bone. Interestingly, female antlers generally lack extensive antler palmation observed in male antlers (Bubenik 1975; Høymork and Reimers 2002; Miller et al. 2021). If male antlers are also targeted by caribou as a nutrient supplement, their more extensive palmation may be more challenging for caribou to fit into their mouths and chew, resulting in different gnawing patterns compared to female antlers. Future studies focused on how migratory caribou modify highly palmated male antlers, which are shed on or near herd mating grounds in the fall (Espmark 1971; Bubenik 1975; Høymork and Reimers 2002; Miller et al. 2021), would provide important insight into how bone consumption by caribou is influenced by antler shape.

Bone modification patterns reveal resource partitioning by caribou and carnivorans, with caribou dominating the consumption of antler resources while carnivorans are the most frequent modifiers of skeletal bones (Figure 4 and Table 2). Yet, beyond this basic partitioning, we also see strong differences in relative resource use; a small minority of antlers on the tundra are left unmodified (by caribou), while carnivorans leave the majority of skeletal bones undamaged. Does this indicate that caribou simply readily encounter antlers on the landscape, while carnivorans are less likely to come across skeletal bones? This seems unlikely based on the consistent presence of skeletal bones in our surveys (Miller et al. 2013), the high mobility of local predators (Mech and Cluff 2011), and the high likelihood that many (if not most) occurrences of adult and sub‐adult skeletal bones likely reflect predator‐caused mortalities. Further, if caribou randomly select any bony resource (antler or skeletal bone) available on the tundra, one would expect far higher occurrences of caribou‐gnawed skeletal bones.

Why are carnivoran tooth marks common but not ubiquitous on skeletal bones of the Coastal Plain? The more limited frequency of skeletal bone modification (compared to caribou‐modified antlers) again points to differences in the importance of bony resources between carnivorans and caribou. Specifically, that caribou are directly targeting and consuming antlers while carnivorans are more often modifying bones as a secondary consequence of their feeding activities. With some notable exceptions (Wald 2011), carnivorans generally target soft tissues or bone marrow and grease associated with bone elements, rather than the bones themselves (Haynes 1983; Faith and Behrensmeyer 2006; Pobiner et al. 2007). Without associated soft tissues, mineralized antlers likely offer less nutritional value to carnivorans such as wolves, wolverines, and bears. Further, during the caribou calving interval, carnivorans have access to abundant soft tissues in the form of caribou calves, which experience high mortality (~25% of calves die between late May and early June; Skoog 1968). Easy access to soft tissues may act to reduce carnivoran bone modification on the Coastal Plain (Haynes 1982, 1983; Fosse et al. 2012). In fact, the high numbers of largely untouched calf carcasses on the calving grounds (Miller et al. 2013) are consistent with our finding that bone destruction does not play a major role in Arctic carnivoran ecology during the caribou calving interval.

Our results illustrate that rodents are not prolific or even common consumers of shed caribou antlers (or skeletal bones) on the Coastal Plain of the Arctic Refuge. The lack of rodent gnawing contradicts the perceived importance of bones as a ubiquitously important resource for rodents (Klippel and Synstelien 2007). Why, then, are Coastal Plain antlers not more highly modified by rodents, and why do skeletal bones show a near lack of rodent modification? If rodent gnawing on antlers and bones functions to alleviate nutritional demands, the limited observed rodent gnawing on the Coastal Plain suggests a sufficient supply of key minerals in dietary resources other than bone. However, sufficient dietary resources would not remove the need for rodents to wear down their incisors. The contributions of our results to these issues are limited, except to indicate that bones and antlers can be virtually unused by rodents, even when readily available on the landscape. The selection of antlers over skeletal bones by rodents is also not immediately clear, though it may simply be a manifestation of the rarity with which rodents modify bones paired with the relative rarity of skeletal bones (compared with shed antlers) on the Coastal Plain. Differences in the ubiquity of rodent gnawing between our results and past studies could also be highlighting taxonomic differences in how rodent groups use bone resources. Notably, porcupines ( *Erethizon dorsatum* ) and tree squirrels (Sciurinae) are both well known to gnaw bones (Klippel and Synstelien 2007; Mori et al. 2018), but are found mostly south of the Coastal Plain (Arctos 2011). Investigations of rodent gnawing on caribou antlers shed in regions with different rodent communities would be useful for evaluating how species composition (and nutrient availability) impacts bone modification by rodents.

As bone is consumed, evidence of past modifications by the same or different individuals or species is destroyed. Could this loss be artificially inflating the perceived dominance of antler gnawing by caribou and systematically biasing the record against rodent and carnivoran modification? We do not think this is likely. First, gnawing by caribou is targeted to distal antler tips; thus progressive gnawing by caribou should not rule out rodent and carnivoran gnawing elsewhere on the bone. Additionally, with tens of thousands of parturient females shedding fresh antlers on the calving grounds each spring, carnivores have ready access to freshly deposited, minimally weathered antlers that are highest in available lipids (Pham et al. 2019). The absence of carnivoran modification across the entire collection indicates that carnivorans rarely, if ever, target antler material on the Coastal Plain. Second, we find rare examples of skeletal bones and antlers with modifications by more than one taxon, indicating that co‐occurrence is possible to detect. This includes four skeletal bones modified by both carnivorans and caribou and 35 antlers gnawed on by caribou and rodents (out of 48 total antlers gnawed by rodents; Table 1). Further, 297 antlers (19%) are largely complete (Gnawing Intensity of 0 or 1) and provide opportunities to record rodent and carnivoran modification in the absence of substantial bone removal by caribou. Combined, the prevalence of caribou modification on antlers appears to be an honest indicator of the role of antlers as a species‐specific dietary supplement and not driven by overprinting previously made modifications by carnivorans or rodents.

**TABLE 1:** Frequency and percentages of antlers and skeletal bones modified by caribou, rodents, and carnivorans (or left unmodified). Column totals for antlers and skeletal bones are slightly higher than the sample size of each bone type because 35 antlers and 4 skeletal bones were modified by multiple taxonomic groups.

Modifier	Antlers (*n* = 1567)	Skeletal bones (*n* = 224)
Caribou	1341 (86%)	12 (5%)
Rodent	48 (3%)	1 (0.5%)
Carnivoran	0 (0%)	91 (41%)
Unmodified	213 (14%)	125 (56%)

**TABLE 2:** Standardized Residuals for chi‐squared test evaluating the frequencies of antlers and skeletal bones modified by caribou, carnivorans, rodents, or left unmodified.

Bone type	Caribou	Carnivoran	Rodent	Unmodified
Antler	25.288391	−25.882353	2.244961	−15.064064
Skeletal bone	−25.288391	25.882353	−2.244961	15.064064

**TABLE 3:** Standardized Residuals for chi‐squared test evaluating the frequency of rodent gnawing for different antler regions. These antler regions were simplified to three (from 10) to accommodate the reduced sample size available of rodent‐gnawed antlers.

	Pedicle	Tine bodies	Tine tips
Rodent‐gnawed	−3.545621	−1.853393	5.399014

Beyond the significance of shed antlers as a nutritional resource for caribou, our data make it clear that ruminants are capable of prolific rates of bone recycling and must be appropriately acknowledged as potentially broadly impactful taphonomic agents. Evaluations of bone as a consumable resource are likely incomplete without considering local ruminant populations. This work also provides an updated toolkit with which to assess ruminants as bone modifiers. Future studies of antler gnawing on the fall mating grounds of caribou, where male antlers are shed, will provide important tests of the ubiquity of antler nutrients in caribou, rodent, and perhaps carnivoran (Wald 2011) diets as they change with season and location. Our findings also highlight the ecological and nutritional value of bones and antlers to local animal populations. In particular, while antler collecting is a popular hobby and has been utilized by multiple institutions for fundraising and other benefits (World Record Academy 2023; Ferraro and Hirst 2024), the scale of their removal should be weighed against the potential negative ecological repercussions.

Why do female caribou have antlers? There remains relatively little consensus regarding the evolutionary drivers of antlers in female caribou. Hypotheses include antlers as weaponry to defend winter foraging patches against conspecifics or to defend against predators (Estes 1991; Schaefer and Mahoney 2001). Female antlers have also been invoked as a mechanism to visually mimic their young male offspring in order to protect them from more aggressive sexually active males (Estes 1991; Reimers 1993; Schaefer and Mahoney 2001). While the theoretical benefits differ in mechanism, all of them focus on the roles that antlers play when still attached to the caribou. Additionally, current hypotheses for the evolution of antlers in female caribou do not consider or meaningfully account for the synchronicity with which migratory females shed their antlers following the birth of their young. This characteristic is without biological correlates among male caribou, which shed their antlers across a more prolonged period following the rut (Sz et al. 2021). Additionally, while antlers are attached to an individual caribou for several months, shed antlers may persist on arctic landscapes for centuries to millennia (Le Moullec et al. 2019; Miller et al. 2021; Miller and Simpson 2022; Miller et al. 2023), likely contributing to the reproductive success of multiple female caribou. While many, or even all, of the above hypotheses may have merit, the benefits of synchronous shedding among migratory female caribou are equally intriguing and may be an important, though overlooked aspect of female antler evolution.

With virtually no interspecies competition for antler resources, our findings illustrate that the nutritional benefits of shed antlers are monopolized by the caribou themselves. Slow Arctic rates of bone weathering and loss (Sutcliffe and Blake 2000; Le Moullec et al. 2019; Miller and Simpson 2022; Miller et al. 2023) further highlight that the landscape‐scale recycling of antler nutrients on the Arctic Coastal Plain is driven by caribou. Previous observations of gnawing by caribou on shed antlers from a caribou calving ground in Norway provided an early indication of their potential nutritional benefits to female caribou (Wika 1982). Antler gnawing by caribou has now been observed in a number of settings, including Norway, and calving grounds in Canada and, now, the USA (Kelsall 1957; Sutcliffe 1973; Wika 1982). The consistency of antler gnawing across many environmental settings, caribou populations, and continents, together with our more systematic quantification presented here, suggests that the consumption of shed antlers could represent a core strategy to supplement caribou diets with key minerals necessary for migration, lactation, and antler regrowth (Sutcliffe 1973; Wika 1982; Barrette 1985; Hutson et al. 2013). Taken together, there is now a growing chorus of evidence indicating that a fundamental benefit of the growth and shedding of antlers by female caribou is the important mineral resources they offer to support annual calving and migration cycles in nutrient‐deficient settings. Further, nearly synchronous shedding of a female's antlers with the birth of her young creates an annually replenished source of calcium and phosphorus that is long‐lasting in Arctic settings (Sutcliffe and Blake 2000; Le Moullec et al. 2019; Miller et al. 2021; Miller and Simpson 2022; Miller et al. 2023). This may even establish a positive feedback loop contributing to the high degree of spatial fidelity of caribou to their calving ground; females in one year gain benefit from previously shed antlers, while newly shed antlers contribute to the herd's reproductive success in subsequent years. Though intriguing, additional research will be important to more explicitly evaluate the dietary and fitness benefits (for both females and their calves) of antler‐derived nutrients. Antlers with labeled calcium and phosphorus would be particularly useful for more directly evaluating the tissues (including those of the calf) that benefit from antler resources. The ecological and evolutionary significance of osteophagy is poorly understood, but the consumption of key nutrients from bones likely serves a variety of biologically consequential roles across mammals and other vertebrates.

## Author Contributions

**Madison Gaetano:** conceptualization (equal), formal analysis (equal), investigation (lead), methodology (equal), visualization (equal), writing – original draft (equal), writing – review and editing (equal). **Eric Wald:** investigation (supporting), resources (supporting), writing – review and editing (supporting). **Patrick Druckenmiller:** investigation (supporting), resources (supporting), writing – review and editing (supporting). **Joshua H. Miller:** conceptualization (equal), data curation (lead), formal analysis (equal), funding acquisition (lead), investigation (supporting), methodology (equal), resources (lead), supervision (lead), writing – original draft (equal), writing – review and editing (equal).

## Conflicts of Interest

The authors declare no conflicts of interest.

## Data Availability

The data that support the findings of this study are openly available and stored with the NSF Arctic Data Center.

## Appendix A

### Methods

#### Mapping Modifications

Antlers are highly variable in shape and anatomy, including the number and location of tines (distinct branches along the antler) and points (terminating projections along a tine; Miller 1986). To establish a standardized antler “map”, we first identified 10 antler regions using five anatomical landmarks (Figure A1). These landmarks are, from proximal‐most (closest to the skull) to distal‐most (farthest from the skull): the base of the antler nearest the pedicle attachment (where the antler joined the skull), brow tine (the tine within several inches distally from the pedicle attachment surface), bez tine (the tine just above the brow tine or, if the brow tine is absent, greater than 3 in. above the pedicle attachment surface), rear tine (located along the posterior side of the antler), and main antler tine (the distal‐most tine emanating directly from the main beam; Figure A1). Because tines may be composed of multiple points, we evaluated the modification status for each one. We numbered the tine tips sequentially, starting with the anteroventral‐most tip, when the antler is held in life position. In order to be counted, a tine must be at least 1 in. long, with a length exceeding its maximum width, as is standard per the Boone and Crockett antler scoring guides (Boone and Crockett Club 2015). Each anatomical landmark was further divided into the “tip” of the feature (the external‐most 1 in. of the landmark) and the “body” (the remainder of the anatomical region; Figure A1). We used inches in our antler measurements in accordance with the Boone and Crockett scoring system, which is a common standard for assessing and comparing antlers (Boone and Crockett Club 2015).

Tine morphologies and lengths are highly variable across different antlers, even within the same species (Lincoln 1992); thus it is not possible to confidently estimate how much bone has been lost when a tine is incomplete. Because of this, our system always refers to the distal‐most 1 in. of the landmark as the “tip”. In this sense, the term “tip” is used as a shorthand for “the end” of the landmark, regardless of whether that anatomical region is complete. This method is useful in situations where modifications are concentrated at bone margins, as opposed to being more randomly distributed across the bone. If a tine terminates in multiple individual points, tips are differentiated with numbers. Thus, a brow tine may include the brow tine body, as well as brow tine tip 1, tip 2, and tip 3. As indicated above, the number 1 tine tip is the anterior‐most and ventral‐most tip in the life position and orientation of the antler. A benefit of our system is that it permits the same standardized data to be taken on incomplete antlers or those originally lacking particular anatomical features. Pairing the “tip” versus “body” differentiation and our five antler landmarks, we mapped each modification (or lack thereof) on each of up to 10 antler regions (Figure A1).

### Caribou Modification (Gnawing) Intensity Scale

To evaluate how taphonomic damage accumulates on individual antlers, we developed a semi‐quantitative scale summarizing the intensity of modification to each antler region. We restricted this analysis to ruminant (caribou) gnawing, which is the dominant modifier of shed antler (see main document Results: Section 5). While we attribute this damage to caribou, our scale works for any ruminant‐gnawed bone. Our scale of gnawing intensity (GI) ranges from 0 to 4: GI 0 indicates an antler without recognizable ruminant modification, though fractures without associated ruminant gnawing may be present (Figure A2). GI 1 (minimal modification intensity) indicates the presence of isolated furrows or punctures by ruminants, but no removal of antler portions via fracturing attributable to ruminant gnawing. GI 2 indicates an antler with one tine that is fractured or removed and has associated gnawing attributable to ruminants along the margins of those fractures. GI 3 indicates an antler with ruminant‐attributable fractures on two or more tines (e.g., brow and bez tines), but retains at least part of the main antler beam distal to the rear tine (or, for antlers that never had rear tines, the region where the antler beam changes direction from a more horizontal to a more vertical beam, which is in the same region as the rear tine). A GI 4 indicates a highly modified antler, including extensive ruminant‐attributed bone removal along the main beam such that the rear tine (or the region of the antler where antler curvature starts to become more vertical) has been removed (Figure A2; Lincoln 1992).

## Appendix B Supplementary Results

As identified in the main text, combining our literature survey and observations of modifications to antlers and skeletal bones from the Arctic Refuge Coastal Plain, we documented 27 distinct bone modification classes. This includes two pits, four punctures, two scores, five furrows, and fourteen fractures Figures A3–A8; (Gaetano et al. 2026). These 27 modification classes represent the full diversity of modifications observed on antlers and bones from the Coastal Plain, as well as modifications known to be generated by taxa living in Arctic settings but not observed on the Arctic Coastal Plain. The frequencies with which each modification class was observed on antlers and skeletal bones are available in Table A1. Each of the modification classes is fully documented in a companion contribution; the Atlas of Arctic Bone Modification (Gaetano et al. 2026). We used letters to indicate modification category (“A” for pits, “B” for punctures, “C” for scores and furrows, “D” for fractures) and numbers to separate class types within that category (see below).

Nine of the 27 modification classes have been described elsewhere in association with tooth and jaw action, including four that are consistent with carnivoran bone gnawing, one attributable to rodent gnawing, and four consistent with previously described gnawing patterns of ruminants, including caribou. Previously described carnivore modifications include rounded pits (A2; documented in Haynes 1982, 1983; Pobiner 2008; Rosell et al. 2019; Haynes and Hutson 2020) and punctures (B3; documented in Haynes 1982, 1983; Pobiner et al. 2007; Pobiner 2008; Rosell et al. 2019; Haynes and Hutson 2020) consistent with carnivoran dentition, rough and isolated furrows extending deep into cancellous bone (associated with the removal of relatively large amounts of bone material in comparison to other described furrows; C5 documented in Rosell et al. 2019; Haynes and Hutson 2020). We also identify fractures consistent with the fraying, splintered textures of “incipient peeling” often described in association with bone modification by bears (but also observed on carcasses modified by lions and hyenas; Rosell et al. 2019; Haynes and Hutson 2020; Gaetano et al. 2026), and “crenulated edges”, which are associated with many species within Carnivora (D8; documented in Pobiner et al. 2007; Pobiner 2008; Haynes and Hutson 2020). We also observed multiple series of shallow, often parallel and overlapping U‐shaped furrows consistent with rodent gnawing (C4; documented in Klippel and Synstelien 2007; Fernández‐Jalvo and Andrews 2016). Two classes of furrows (C1.3 and C3, Figure 1c,d) and one fracture class (D3, Figure 1b) were consistent with previously identified bone modification by ruminants (Sutcliffe 1973; Wika 1982; Cáceres et al. 2011; Rassadnikov 2021). One class of punctures (B4; ‘keyhole punctures’ documented in Tokunaga 1936 and Cáceres et al. 2011) was not observed on the Arctic Coastal Plain but has been previously identified as a characteristic damage pattern generated by cervids consuming bone.

Below, we summarize seven newly recognized classes of ungulate‐generated bone modification that are being first explored here and a companion resource (the Atlas of Arctic Bone Modification, Gaetano et al. 2026). We also formalize the description of 11 classes of bone modification we consider to be ambiguous in origin, being consistent with bone‐environment interactions (e.g., antlers rubbing on rocks during develveting, trampling, etc.), or interactions between bone and a potentially broad suite of ruminants, raptors, or mammalian carnivorans. Additional information and example photographs can be found in the Atlas (Gaetano et al. 2026). The Atlas also documents overall generalities and consistencies of bone modifications made by ruminants, carnivorans, and rodents, as well as ways of distinguishing them visually.

### Pits and Punctures

We observed one pit and two puncture classes that have not been previously described. The previously undescribed pit class (A1, Figure A4; Gaetano et al. 2026), is circular in map‐view, generally several millimeters to a centimeter in diameter, and has a smooth, unbroken margin. These pits are deepest at the center and become shallower closer to the margins, are typically confined to the outer cortical bone and may have a rough interior. We do not attribute a likely modifier to A1, as we find its appearance inconsistent with descriptions of tooth and bite‐derived modification (Pobiner et al. 2007; Pobiner 2008; Fosse et al. 2012; Rosell et al. 2019). While the origins of this feature are ambiguous, we suggest that it may derive from pre‐shedding “marring,” which can occur when a cervid rubs their antlers against hard surfaces in the environment, including trees, other antlers, rocks, and hard substrates (Olsen 1989; Jin and Shipman 2010).

The two newly described classes of puncture, which were primarily observed on antlers (Table A1), are distinct in that their margins are straight and they typically occur in clusters of two or more. The first (“B1”, Figure 1e; Gaetano et al. 2026) is a short (longest axis < 5 mm) linear puncture that creates a shallow incision within the bone. The depths of these features are generally inconsistent across their lengths and the deepest regions are generally towards their centers. B1 punctures are typically restricted to cortical bone, but can extend into underlying cancellous bone at their deepest point. The second puncture class (B2, Figure 1f; Gaetano et al. 2026) is readily distinguished from other features by its straight edges, which generally have a triangular form in map view. In three dimensions, they are roughly irregular tetrahedrons, with their apices extending into the bone. Both puncture classes are visually distinct from circular and/or conical punctures typically characteristic of bone modification from carnivorans (bear, wolf; Fosse et al. 2012; Fernández‐Jalvo and Andrews 2016; Rosell et al. 2019), and are instead consistent with the roughly tetrahedral cusps of caribou premolars and molars. Additionally, while punctures produced by carnivorans often include shattered bone material pressed into the inner regions of the punctures (Pobiner 2008), B2 punctures do not contain bone fragments within their voids. While the angular nature of B2 punctures is somewhat reminiscent of rare and small (maximum length ≈1.8 mm) triangular pits found on some skeletal bones attributed to red fox ( *Vulpes vulpes* ; Arilla et al. 2019), B2 punctures are larger (maximum length ≈3.8 mm) and pressed deeper into the bone. Additionally, while fox pits form equilateral triangles with three visually distinct edges, the margins of B2 punctures are more variable in their visual sharpness, with the clearest margin generally found along one of the longest edges of the isosceles (where the puncture is also the deepest; Figure A5). In addition to clear morphological differences between fox pits and B2 punctures, there is also no co‐occurrence between B2 punctures and any recognized class of carnivoran modification (B3, C5, and D7) on the same antler (which would be expected if B2 punctures had been made by foxes; Arilla et al. 2019). However, finding signs of foxes gnawing on antlers would be surprising, as foxes are known to ignore antlers entirely (Gambín et al. 2017). Across all occurrences of B1 and B2 punctures (*n* = 589 and 365, respectively), 10% and 15% of those occurrences co‐occur with known caribou modifications (Table A2). Based on similarity in shape and dimensions between B2 punctures and ruminant teeth, we attribute these marks to caribou. The overall shape and dimensions of linear B1 punctures are also consistent with caribou cheek teeth and are likely incompletely formed B2 punctures.

#### Scores and Furrows

We observed two previously described classes of isolated scores and one novel class of overlapping furrows (Table A3). Isolated scores and furrows can occur in groups (generally not exceeding ~5) and occasionally bisect one another. However, the areas of overlap for both scores and furrows are generally limited relative to the total surface area of the feature. Additionally, the margins of each individual score or furrow generally remain distinguishable from each other. The two isolated scores (C1.1 and C1.2, Figure A2; Gaetano et al. 2026) are both shallow, linear features that are generally confined to the surfaces of cortical bone. C1.1 scores are extremely shallow and have minimal variation in depth when assessed visually (using either a hand lens or microscope). Further, when running a finger along C1.1 scores, it can be challenging to feel any variation in depth. Instead, C1.1 scores are often most readily identified by their lighter color, relative to surrounding, unmodified bone (due to exposure of bone below the outermost layer). C1.2 scores extend slightly deeper into the cortical bone than C1.1 scores and have more variability in the depth of individual scores, though C1.2 scores are generally still confined to cortical bone. C1.2 scores may appear rough, with visible U‐shaped cross sections. Both C1.1 and C1.2 scores are consistent with modifications that develop while the antler is attached to the skull and in use (i.e., during contact with other antlers, trees and other hard surfaces pre‐shedding; Olsen 1989; Jin and Shipman 2010).

Overlapping furrows are a hallmark of ruminant gnawing modification (cataloged in this text as “C3” furrows, Figure A7; Sutcliffe 1973; Wika 1982; Cáceres et al. 2011; Hutson et al. 2013), but the furrows described here (C2 furrows, Figure A7; Gaetano et al. 2026) expose less cortical bone than previously described furrows. C2 furrows are chaotic amalgamations of individual furrows that are tightly spaced and highly overlapping. C2 furrows are U‐shaped in cross‐section, but their highly overlapping nature can make it difficult to distinguish individual furrows. While this amalgamation removes some of the outer cortical bone, the depth of removal is not uniform. Further, some regions of cortical bone within the borders of the feature may be unmodified, resulting in a bumpy and rough texture (both visually and tactilely). This inconsistency in feature depth is a main differentiation between C2 and previously described C3 furrows (Figure A7; Gaetano et al. 2026), which have more consistent depths of bone removal. Across all occurrences of C2 furrows (*n* = 381), 10% co‐occurred with known caribou modifications (Table A2). C2 furrows are visually similar to those described (Cáceres et al. 2011) from deer‐modified bones (Gaetano et al. 2026), and distinguishable only by their more limited removal of cortical bone. We attribute C2 furrows to ruminant (caribou) modification and interpret C2 furrows as the initial stages of modification that lead to C3 furrows.

#### Fractures

We document 14 unique classes of fracture: D1, D2, D3, D4.1, D4.2, D5.1, D5.2, D5.3, D5.4, D6, D7, D8, D9, and D10 (Figure A8; Gaetano et al. 2026). Of these, we attribute five to ruminant modification (D1, D2, D3, D4.1, and D4.2), and one to carnivore modification (D7). The rest are unattributed to a specific modifier. The first two fracture classes are characterized by fracture margins that run straight, perpendicular to the body of the broken tine (D1) or fracture margins that run at an angle relative to the broken tine (D2). The fractures may have some bone splintering along the outer edge, but the fracture surface is generally continuous and without extensive bone splintering. The outer edge is often compressed, becoming increasingly thin towards the outer edge of the fracture. Bone that remains along the boundary of the fracture surface is often associated with previously described modification features attributable to ruminant modification, including overlapping furrows (C3) and deep, U‐shaped furrows (C1.3; Sutcliffe 1973; Wika 1982; Cáceres et al. 2011; Hutson et al. 2013). D1 and D2 fractures co‐occur (are found on the same antler element) with recognized classes of ruminant modification (C3 and C1.3 furrows) in 58% (426/735) and 80% (1064/1336) of occurrences, respectively (Table A2). Due to this consistent association between well‐studied ruminant modification and both D1 and D2 fractures, we attribute both fracture classes to ruminant modification.

D4.1 and D4.2 are characterized by fracture surfaces composed of individual, distally thinning bone splinters that are largely or entirely devoid of interior cancellous bone (i.e., splinters are mostly composed of cortical bone). Splinters can range in length from ~1 cm to upwards of 3–4 cm. Emanating from more complete bone, these bone splinters may form a partial arc along the outer edge of the antler tine's (or beam's) transverse cross‐section (D4.1; Figures 1j and A4), or these bone splinters may entirely ring that transverse cross‐section, creating something similar to a crown (D4.2; Figures 1k and A4). D4.1 and D4.2 are closely associated with C3 and C1.3 classes of ruminant gnawing (161/169 and 43/46 occurrences, respectively; Table A2). Based on this close association, we attribute both D4.1 and D4.2 to ruminant gnawing.

The remaining fracture classes, D5.1, D5.2, D5.3, D5.4, D6, D8, D9, and D10 are not associated with pits, punctures, furrows, or fractures that have been previously attributed to gnawing or biting by known modifiers. As such, we do not attribute them to specific modifiers. Instead, these classes are most consistent with modifications previously observed prior to or following shedding, when antlers rub against one another or the surrounding environment, including trees and hard substrates (Olsen 1989; Jin and Shipman 2010). Broken margins of D5.1 fractures appear as crescent or U‐shaped notches taken out of the remaining bone (Figure A8; Gaetano et al. 2026). Though D5.1 is confined to cortical bone and is associated with the removal of relatively little bone material, we group them here because they modify the shapes of antler margins and are likely to arise from similar force‐loading as more extreme classes of bone fracture (Pobiner et al. 2007). D5.2 is also a crescent‐shaped fracture, but is differentiable because it extends into the cancellous bone (Figure A8; Gaetano et al. 2026). While D3 fractures are also U‐shaped, both D5.1 and D5.2 fractures can be distinguished from D3 fractures in that they are not associated with furrows or compression along the surface of the bone, aside from the fracture margin (Gaetano et al. 2026). D5.1 and D5.2 fractures also generally have smooth edges, while D3 fractures have rough margins. D5.3 and D5.4 are relatively straight fractures along the surface of an antler, though D5.3 is associated with minimal variation in depth across the surface of the feature, while D5.4 is highly angled with broad variation in depth throughout the feature (Figure A8; Gaetano et al. 2026). D6 (Figure A8; Gaetano et al. 2026) is characterized as having a beveled edge, often sloping down at an angle that is parallel to the antler tine, with a narrow ridge of raised outer bone material at the base of the feature that runs parallel to the long‐axis of the antler tine (consistent with beveled fracturing described previously; Olsen 1989; Jin and Shipman 2010; Gaetano et al. 2026). D8 fractures (Figure A8; Gaetano et al. 2026) have crenulations, or “notched,” patterns along the fracture edge, a texture often observed on bones following modification by carnivorans (Pobiner et al. 2007; Rosell et al. 2019; Haynes and Hutson 2020). However, we also note that similar “notched” patterns could arise from interactions with the environment, such as being stepped upon by hooves. While we do not dispute that this feature may indicate modification by carnivorans, we require the presence of additional classes of carnivoran modification (including B3 punctures, C5 furrows, or D7 fractures), to identify a bone as having been modified by a carnivoran. D9 and D10 fractures (Figure A8; Gaetano et al. 2026) also include crenulated, notched edges, but crenulated patterns are less consistent along D9 fractures relative to D8, and less so along D10 fractures relative to D9.

We found no instances of keyhole fractures (Figure 1a; Tokunaga 1936; Cáceres et al. 2011) among the 9,129 occurrences of modifications to bones and antlers from the Coastal Plain of the Arctic Refuge. This absence is notable because keyhole fracturing was likely the first class of cervid bone modification that was formally described and has been thoroughly documented in both red and fallow deer ( *Cervus elaphus* , *Dama dama* ; Cáceres et al. 2011). Keyhole fractures are also one of three diagnostic modification “stages” of cervid modification and have been considered an intermediary between overlapping furrows (cataloged in this work as C3) and forked fractures (cataloged in this work as D3; Sutcliffe 1973; Cáceres et al. 2011). Despite frequent observations of both overlapping furrows (C3) and forked fractures (D3) in our dataset, we do not observe this inferred transitional state (i.e., keyhole fractures) between the two. Keyhole fractures were first observed on the limb bones of an unknown cervid species from Quaternary deposits of the Japanese Riukiu Islands and appear to form when chewing is concentrated on, and possibly expands, vascular and/or neural foramena of bones (Tokunaga 1936; Kawamura et al. 2016). The lack of such foramena or fossae on antlers may prevent this classic modification class from forming. However, it is notable that we also do not observe this modification class on modified skeletal bones from the Arctic Coastal Plain. Consistency in the teeth and jaws among cervids suggests that caribou should be able to generate keyhole modifications and that their absence is due to the clear focus of bone gnawing on antlers; caribou may simply not be gnawing on skeletal bones long enough to generate the keyholes. Additionally, the distal points of antlers, where gnawing by caribou is focused, are primarily composed of spongy cancellous bone, which may not be structurally strong enough to support keyhole fractures before simply breaking off in the mouth of the modifier.

**TABLE A1:** Modification classes and attributed modifying agent (ruminant, rodent, carnivoran, unknown) for the 9,129 occurrences of modifications on antlers and skeletal bones. Modification classes described for the first time here (and the Atlas of Arctic Bone Modification; Gaetano et al. 2026) are noted with an asterisk. A brief justification for the assigned modifier is provided here. A more complete assessment is provided in the text and the Atlas of Arctic Bone Modification (Gaetano et al. 2026).

Modification class	Frequency		Attributed modifier	Modifier justification
	Antler	Skeletal bone		
*Pits*				
A1	90	2	Unattributed	Consistent with non‐tooth based modification, which can accumulate prior to or following shedding (in the case of antlers), typically arising from interactions with the surrounding environment (i.e., via contact with hard surfaces like rocks, trees, hooves; Olsen 1989; Jin and Shipman 2010)
A2	0	42	Carnivoran	Consistent with tooth marks produced by carnivorans while biting or gnawing on bones (Pobiner et al. 2007; Pobiner 2008; Fosse et al. 2012; Arilla et al. 2019; Rosell et al. 2019; Haynes and Hutson 2020; Keyes et al. 2020)
*Punctures*				
B1*	589	3	Ruminant	Consistent with cusp shape of ruminant cheek teeth and inconsistent with rounded punctures typical of known carnivoran modifiers. Also lacking fragmented bone sometimes associated with carnivoran punctures (Pobiner et al. 2007; Fernández‐Jalvo and Andrews 2016; Rosell et al. 2019)
B2*	365	0	Ruminant	See justification for B1 punctures. Distinct from the triangular fox puncture photographed in Arilla et al. (2019), as B2 punctures form obtuse isosceles triangles, are larger in size and extend further into bone material
B3	0	60	Carnivoran	Consistent with modifications observed in association with consumption by carnivorans e.g., bears, wolves, hyenas, foxes; (Pobiner et al. 2007; Pobiner 2008; Fosse et al. 2012; Arilla et al. 2019; Rosell et al. 2019; Haynes and Hutson 2020; Keyes et al. 2020)
B4	0	0	Ruminant	Keyhole puncture interpreted as an intermediate ruminant gnawing stage, occurring between overlapping furrows (C3) and U‐shaped fractures (D3; Cáceres et al. 2011)
*Scores*				
C1.1	139	4	Unattributed	No modifier assigned. In the case of isolated scores, it is challenging to differentiate ruminant, carnivoran (or other) modifiers, or modification derived from tooth action, from those originating via interactions with hard elements in the surrounding environment (e.g., rocks). The teeth of ruminants and carnivorans, or talons and beaks of raptors can produce visually similar isolated scores (Pobiner et al. 2007; Reeves 2009; Hutson et al. 2013; Rassadnikov 2021). Trampling by hooves, or rubbing against other antlers, trees, rocks, or other hard surfaces in the environment can also produce “scratches” or scores (Haynes and Stanford 1984; Olsen 1989; Jin and Shipman 2010; Courtenay et al. 2020)
C1.2	1,231	56	Unattributed	No modifier is assigned. See justification for C1.1 scores
*Furrows*				
C1.3	126	0	Ruminant	Furrows are consistent with previously documented classes of caribou modification ( *Rangifer tarandus* ; Sutcliffe 1973; Wika 1982)
C2*	381	0	Ruminant	Similar, though less developed (lacking the consistent and even removal of outer bone), to C3 modification (see below), which is consistent with modifications from ruminant gnawing (Cáceres et al. 2011; Hutson et al. 2013)
C3	2,461	9	Ruminant	Consistent with modification by cervids (ruminants), including *Dama dama* and *Cervus elaphus* , gnawing on bone (Cáceres et al. 2011; Hutson et al. 2013)
C4	78	1	Rodent	Consistent with well‐studied characteristics of rodent modification (Klippel and Synstelien 2007; Pokines et al. 2017)
C5	0	33	Carnivoran	Consistent with well‐studied characteristics of carnivoran (e.g., bears, wolves, hyenas; Pobiner et al. 2007; Fosse et al. 2012; Kawamura et al. 2016; Arilla et al. 2019; Rosell et al. 2019) modification
*Fractures*				
D1*	735	0	Ruminant	Bone along fracture margins is compressed, which is consistent with of ruminant modification (Sutcliffe 1973). D1 are often found co‐occurring with C3 or C1.3 furrows (Table A2), which are hallmarks of ruminant gnawing (Sutcliffe 1973; Wika 1982; Cáceres et al. 2011)
D2*	1,336	0	Ruminant	See justification for D1 fractures
D3	288	0	Ruminant	Consistent with previously described modification by ruminants (Sutcliffe 1973; Cáceres et al. 2011; Hutson et al. 2013)
D4.1*	169	0	Ruminant	Nearly always associated with C3 and C1.3 furrows (95% of occurrences), which were previously established as diagnostic features of ruminant bone modification (Cáceres et al. 2011)
D4.2*	46	0	Ruminant	Nearly always associated with C3 and C1.3 furrows (93% of occurrences), which were previously established as diagnostic features of ruminant bone modification (Cáceres et al. 2011)
D5.1	7	0	Unattributed	Consistent with non‐tooth derived modification, which accumulates prior to or following shedding, arising from contact with other antlers, trees, rocks, etc. within the environment (Bowyer 1983; Olsen 1989; Jin and Shipman 2010)
D5.2	6	0	Unattributed	See justification for D5.1 fractures
D5.3	184	6	Unattributed	See justification for D5.1 fractures
D5.4	224	36	Unattributed	See justification for D5.1 fractures
D6	269	0	Unattributed	See justification for D5.1 fractures
D7	0	5	Carnivoran	Consistent with “peeling” modification produced by carnivores during carcass consumption (Rosell et al. 2019; Haynes and Hutson 2020)
D8	0	31	Unattributed	Consistent with modification observed during predation and carnivoran bone modification (Pobiner et al. 2007; Arilla et al. 2019; Rosell et al. 2019). However, we do not assign a modifier because visually similar fractures may reasonably occur due to damage caused by interactions with the environment (e.g., antler rubbed on a rock)
D9	0	108	Unattributed	May include individual or isolated crenulations along a fracture margin, but not with enough regularity to be identified as a crenulated edge.
D10	0	9	Unattributed	See justification for D9 fractures

**TABLE A2:** Co‐occurrence between previously documented classes of bone modification by ruminants (identified here as C1.3, C3, and D3) with those described here (B1, B2, C2, D1, D2, D4.1, D4.2). Co‐occurrence is defined as classes of modification that occur on the same antler region (e.g., on the same antler tine).

Modification class	Total occurrences	Co‐occurrences with previously documented classes of ruminant modification	Co‐occurrence percentage
B1	589	56	10%
B2	365	54	15%
C2	381	40	10%
D1	735	426	58%
D2	1,336	1,064	80%
D4.1	169	161	95%
D4.2	46	43	93%

**TABLE A3:** Co‐occurrence of D8, D9, and D10 fractures with previously documented classes of carnivoran modification (identified here as A2, B3, C5, and D7). Co‐occurrence is defined as modification that occurs on the same bone element.

Modification class	Total occurrences	Co‐occurrence with previously documented classes of carnivoran modification	Co‐occurrence percentage
D8	31	19	61%
D9	108	44	41%
D10	9	5	56%
